# Integrating Mechanisms of Exacerbated Atrophy and Other Adverse Skeletal Muscle Impact in COPD

**DOI:** 10.3389/fphys.2022.861617

**Published:** 2022-06-03

**Authors:** Tanja Taivassalo, Russell T. Hepple

**Affiliations:** ^1^ Department of Physiology and Functional Genomics, University of Florida, Gainesville, FL, United States; ^2^ Department of Physical Therapy, University of Florida, Gainesville, FL, United States

**Keywords:** muscle atrophy, mitochondria, neuromuscular junction, mitochondrial permeability transition, aryl hydrocarbon receptor, smoking

## Abstract

The normal decline in skeletal muscle mass that occurs with aging is exacerbated in patients with chronic obstructive pulmonary disease (COPD) and contributes to poor health outcomes, including a greater risk of death. There has been controversy about the causes of this exacerbated muscle atrophy, with considerable debate about the degree to which it reflects the very sedentary nature of COPD patients vs. being precipitated by various aspects of the COPD pathophysiology and its most frequent proximate cause, long-term smoking. Consistent with the latter view, recent evidence suggests that exacerbated aging muscle loss with COPD is likely initiated by decades of smoking-induced stress on the neuromuscular junction that predisposes patients to premature failure of muscle reinnervation capacity, accompanied by various alterations in mitochondrial function. Superimposed upon this are various aspects of COPD pathophysiology, such as hypercapnia, hypoxia, and inflammation, that can also contribute to muscle atrophy. This review will summarize the available knowledge concerning the mechanisms contributing to exacerbated aging muscle affect in COPD, consider the potential role of comorbidities using the specific example of chronic kidney disease, and identify emerging molecular mechanisms of muscle impairment, including mitochondrial permeability transition as a mechanism of muscle atrophy, and chronic activation of the aryl hydrocarbon receptor in driving COPD muscle pathophysiology.

## Introduction

Chronic obstructive pulmonary disease (COPD) patients are often affected by more severe limb skeletal muscle atrophy than is typical of normal aging (Corlateanu et al., 2016), with mounting evidence that, as with other comorbidities, muscle atrophy exacerbates patient outcomes (Marquis et al., 2002; Jones et al., 2015; Attaway et al., 2021). Whilst the incidence of muscle atrophy may be somewhat higher in male (15–38%) than female (16–25%) COPD patients, both male and female patients may exhibit muscle atrophy (Anderson et al., 2017). Amongst the first studies to establish that the degree of skeletal muscle atrophy is greater in patients with COPD than normal aging was the work of van den Borst and colleagues (van den Borst et al., 2011). In that seminal study they examined age-matched men (70–79 years) that comprised 260 patients with COPD, 157 smoking controls, 866 former-smoking controls, and 891 never-smoking controls. The primary finding was that COPD patients and current smokers without COPD had lower muscle mass (determined by dual x-ray absorptiometry [DEXA]) than age-matched non-smoking study participants (van den Borst et al., 2011). Similar findings were reported in a study of 5,082 participants (mean age = 69 years; 56% female) from the Rotterdam study, where patients with chronic airway diseases had a lower appendicular skeletal muscle mass index as measured by DEXA than age-matched healthy controls (Benz et al., 2021). The degree to which there may be differences in the biological processes underlying the muscle atrophy occurring with aging, vs. that which is superimposed on the former by COPD and its predisposing factors such as long-term smoking, remains a work in progress. Amongst the goals of this review is to discuss similarities and differences in the phenotypic manifestations of muscle with normal aging vs. that seen in COPD, and to provide some consideration for the biological mechanisms that may account for these differences. It must be stressed, however, that this review merely offers one perspective on this evolving issue and that further research is needed.

**FIGURE 1:**
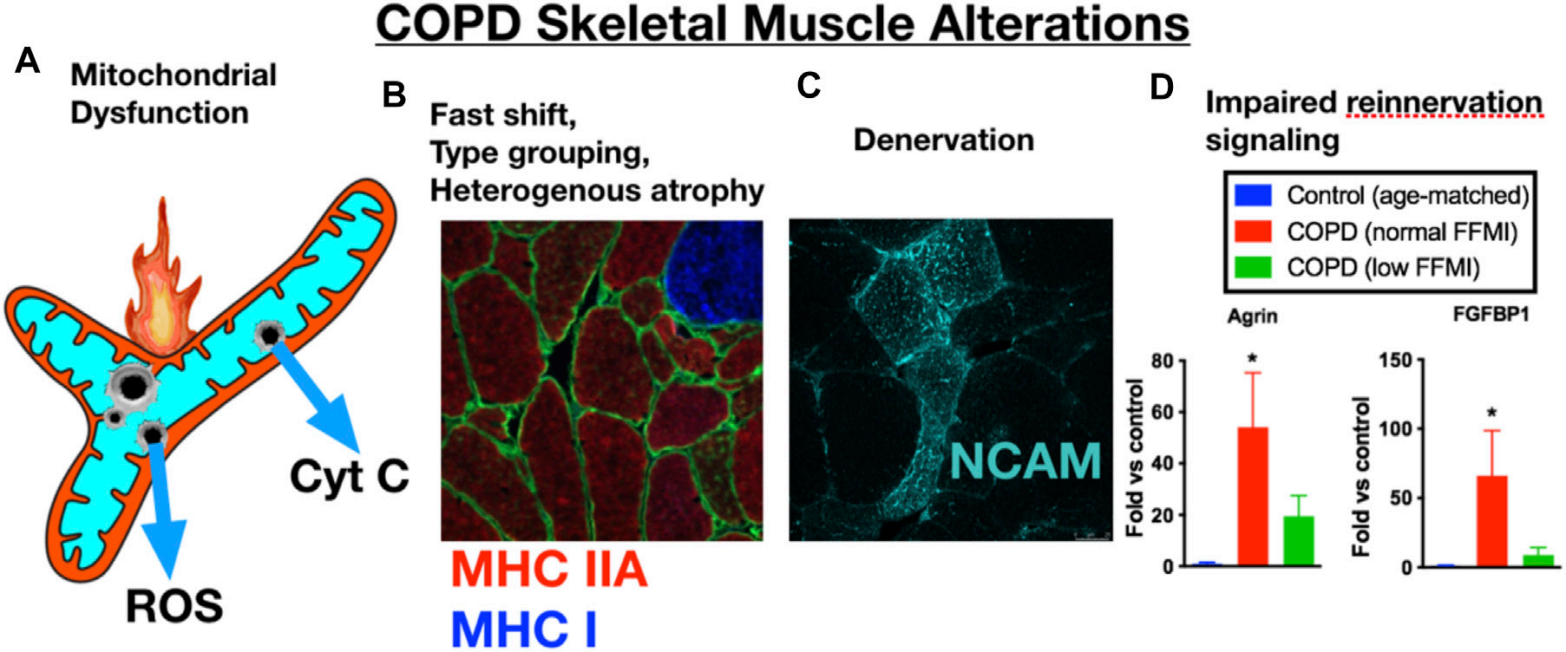
COPD patient limb muscle is characterized by a variety of alterations that include **(A)** mitochondrial dysfunction [reduced respiratory capacity, elevated reactive oxygen species (ROS), and evidence of an increased frequency of mitochondrial permeability transition events causing release of cytochrome c, **(B)** a fast fiber shift and fast fiber type grouping indicative of recurring denervation-reinnervation events, **(C)** accumulation of muscle fibers expressing markers of denervation (e.g., neural cell adhesion molecule (NCAM)], and **(D)** a transcriptional profile suggesting an impaired reinnervation response. Images and data in panels **(B–D)** are adapted from (Kapchinsky et al., 2018). MHC IIA = type IIa fibers; MHC I, type I fibers; FFMI, fat free muscle index; FGFBP1, fibroblast growth factor binding protein 1 (promotes collateral sprouting of neighboring motoneurons to promote reinnervation).

In addition to skeletal muscle atrophy, there are many other manifestations of adverse limb muscle impact in COPD (summarized in Figure 1). These include reduced endurance/lower fatigue resistance (Allaire et al., 2004; Bachasson et al., 2013; van den Borst et al., 2013), altered mitochondrial function (Slebos et al., 2007; Picard et al., 2008; Puente-Maestu et al., 2009a; Puente-Maestu et al., 2009b), an increase in fast fiber abundance (Gosker et al., 2007), increased abundance of fibers expressing multiple myosin heavy chains (MHCs) simultaneously (so-called MHC co-expressing fibers) (Kapchinsky et al., 2018), and an accumulation of very small muscle fibers (Gosker et al., 2002) that exhibit features of prolonged denervation (Kapchinsky et al., 2018). Interestingly, in COPD patients with low muscle mass there is a blunted reinnervation transcriptional response, suggesting that failed reinnervation is a likely driver of the aggravated muscle atrophy in COPD (Kapchinsky et al., 2018). In view of the association between severity of skeletal muscle alterations, including atrophy, and poor health outcomes in COPD patients (Gosker et al., 2007; van de Bool et al., 2016; Attaway et al., 2021), this review focuses upon our current understanding of the mechanisms contributing to the adverse skeletal muscle impact in COPD patients.

## Mitochondrial Impact in Chronic Obstructive Pulmonary Disease Skeletal Muscle

Mitochondria serve numerous roles in the cell and are a key point of homeostatic regulation. For example, not only are mitochondria integral to ATP generation and ROS signaling in skeletal muscle (Hood et al., 2019), recent evidence also indicates that mitochondria are directly involved in mediating muscle atrophy through the process of mitochondrial permeability transition (Burke et al., 2021) (see below for more on this point). Mitochondria are enriched in skeletal muscle, particularly in so-called oxidative muscle fibers (type I, type IIa), including dense mitochondrial accumulation at the neuromuscular junction in both the pre-synaptic motoneuron terminals and in the region beneath the acetylcholine receptors on the muscle fiber (Anagnostou and Hepple, 2020). For these reasons, alterations in mitochondrial function with COPD are likely to be involved in various aspects of the adverse skeletal muscle impact seen in COPD, including muscle atrophy, impaired fatigue resistance, and denervation.

Consistent with an important role of mitochondria in COPD skeletal muscle impairment, a variety of mitochondrial alterations are commonly reported in studies examining skeletal muscle in COPD patients. Examples of these alterations include a reduced activity of mitochondrial enzymes, reduced muscle respiratory capacity, and elevated mitochondrial reactive oxygen species emission (ROS) (Puente-Maestu et al., 2009a; D'Agostino et al., 2010; Naimi et al., 2011; van den Borst et al., 2013). It is important to point out that these mitochondrial alterations exceed those already occurring with normal aging in skeletal muscle, and the reader is directed to a recent review of this latter topic (Alway et al., 2017). Regarding the nature of the changes in mitochondrial function in COPD skeletal muscle, it has been questioned whether the alterations seen represent an impairment in organelle-specific function or whether they are a consequence of the observed shift towards a greater abundance of fast twitch fibers in COPD patients (Picard et al., 2008), noting that mitochondria in fast twitch muscle are less abundant, have higher ROS emission, and have higher calcium retention capacity (Picard et al., 2012). Importantly, however, not all COPD patients exhibit a fast fiber shift (Gosker et al., 2007) and no prior studies have compared mitochondrial function between patients who exhibit a fast fiber shift vs. those who do not exhibit this fast fiber shift. Such an analysis will be important in further understanding the potential link between fiber type shift and mitochondrial function alterations in COPD muscle. In support of an organelle-specific defect, Puente-Maestu and colleagues reported a sensitization of mitochondria to undergo permeability transition and this was associated with increased cytochrome c release from incubated isolated mitochondria of COPD limb skeletal muscle (Puente-Maestu et al., 2009b), noting that mitochondrial permeability transition is a well-established mechanism of tissue dysfunction in various organs (Bauer and Murphy, 2020) and has been proposed to play a role in aging skeletal muscle (Hepple, 2016).

**FIGURE 2:**
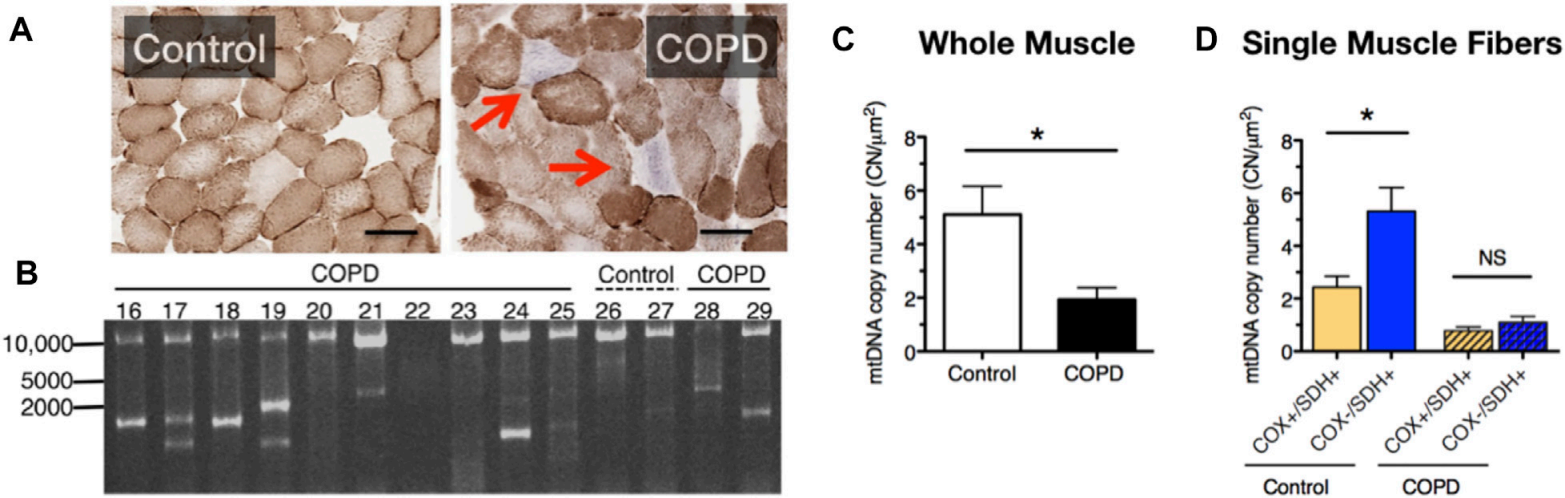
COPD patient muscle evidences **(A)** an increased accumulation of muscle fibers with respiratory impairment (fibers indicated by the red arrows lack cytochrome oxidase activity), **(B)** accumulation of mtDNA deletion mutations, **(C)** reduced mtDNA copy number, and **(D)** an impaired up-regulation of mtDNA copy number in response to energetic stress in muscle fibers with respiratory impairment. Images and data in panels **(B–D)** are adapted from (Konokhova et al., 2016).

To further test for manifestations of mitochondrial dysfunction in COPD limb muscle, in one of our prior studies we used a histochemical diagnostic approach to identify muscle fibers with severe mitochondrial respiratory capacity impairment and observed a markedly elevated abundance of muscle fibers exhibiting depletion of mitochondrial complex IV activity but relatively normal (or elevated) mitochondrial complex II activity in vastus lateralis muscle biopsies of COPD patients (Konokhova et al., 2016). This phenotype of selective complex IV deficiency (with or without elevated complex II activity) is characteristic of muscle fibers harboring a high burden of mitochondrial DNA (mtDNA) mutations (Bua et al., 2006; Rocha et al., 2015), and occurs with normal aging and some forms of primary mitochondrial disease, amongst other conditions (Rak et al., 2016). The basis of this diagnostic histochemical assay is that complex IV contains 3 mtDNA-encoded subunits, whereas complex II is entirely nuclear encoded; hence, muscle fibers (or segments therein) with high levels of mutations affecting mtDNA regions encoding subunits of complex IV will exhibit preferential depletion of complex IV activity (see blue fibers denoted by arrow in Figure 2A). Indeed, in addition to the higher levels of complex IV deficient muscle fibers, we also observed a high frequency of mtDNA deletions (Figure 2B) and reduced mtDNA copy number (Figure 2C) in whole muscle homogenates of COPD locomotor muscle. Furthermore, those patients with mtDNA deletion mutations had higher levels of oxidative damage, higher smoking pack years, and a lower maximal exercise capacity (Konokhova et al., 2016).

It is noteworthy that muscle fibers with complex IV deficiency typically exhibit a compensatory upregulation of mitochondrial biogenesis and mtDNA copy number in a futile attempt to restore the bioenergetic capacity of the muscle fiber (futile because the high burden of mutated mtDNA templates precludes biogenesis of properly functioning mitochondria). Whereas in age-matched control subjects fibers with complex IV deficiency and normal or elevated complex II activity (Cox-/SDH+) exhibited greater than 2-fold higher levels of mtDNA copy number compared to normal muscle fibers (Cox+/SDH+), this was not seen in COPD patient muscle (Figure 2D) and this phenotype was associated with an impaired translation of mtDNA transcription factor A (TFAM) (Konokhova et al., 2016). As such, these results identify an impaired mtDNA replication and mitochondrial biogenesis response to severe energetic stress in COPD muscle, a finding that likely contributes to the impaired adaptive response to aerobic exercise training (a milder form of energetic stress that induces mitochondrial biogenesis) that is often seen in COPD patients (Tenyi et al., 2018; Latimer et al., 2021). Related to this, because abnormal mitochondrial function with mtDNA mutation accumulation requires a high fraction of mutated:normal mtDNA genomes [estimated to be >80% (Sciacco et al., 1994)], the low absolute level of mtDNA copy numbers in COPD locomotor muscle means that fewer absolute numbers of mtDNA genomes need to be mutated for mitochondrial dysfunction to ensue within individual muscle fibers in COPD (Konokhova et al., 2016).

Further to the relevance of specific mitochondrial enzymatic abnormalities in COPD, a recent study using a mouse model of pulmonary emphysema observed a down-regulation of subunit c of complex II (SDH) that was associated with lower muscle respiratory capacity and greater muscle fatigability. In addition, genetic gain of function experiments to rescue complex II in mice with emphysema improved muscle respiratory capacity and reduced fatigability (Balnis et al., 2021). Amongst the most important contributions from this recent work is that it helps us begin to parse out the contribution of COPD pathophysiology vs. that which is secondary to long-term smoking independent of lung disease. Specifically, the investigators used a model of emphysema that occurs consequent to genetic upregulation of IL-13 in Club cells, and which produces not only hallmark features of lung pathology seen with COPD, but also skeletal muscle features such as atrophy and impaired muscle respiratory capacity seen in patients (Balnis et al., 2020a). It will be interesting in future studies to compare the nature of the muscle alterations seen in this and other lung disease models to that occurring with smoking, to more precisely address the potential for interactions between COPD pathophysiology and smoking-induced alterations in skeletal muscle.

**FIGURE 3:**
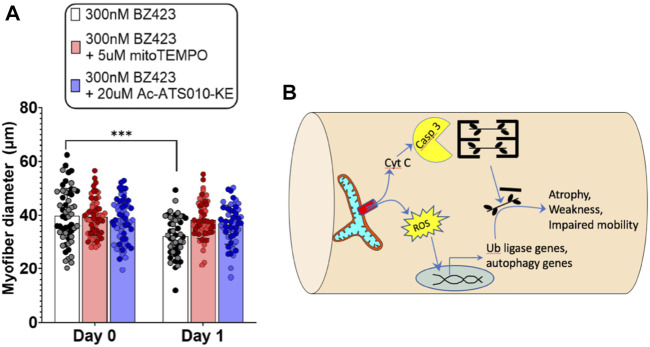
Recent evidence identifies mitochondrial permeability transition (MPT) as a mechanism that induces skeletal muscle atrophy in a mitochondrial ROS- and Caspase 3-dependent manner (Burke et al., 2021). **(A)** Single FDB muscle fibers from adult mice treated with Bz423 (to induce MPT) for 24 h demonstrated significant atrophy that could be prevented by inhibiting mitochondrial ROS using mitoTemo or by inhibiting caspase 3 using Ac-ATS010-KE (figure panel adapted from (Burke et al., 2021)). **(B)** Schematic illustration showing how MPT-induced release of cytochrome C leads to activation of caspase 3 that in turn leads to cleavage of actin from the muscle cross-bridges, and release of ROS that activates ubiquitin ligase and autophagy signaling to promote muscle atrophy.

Despite mitochondria frequently being implicated in muscle atrophy processes (Romanello and Sandri, 2021), no prior studies have directly assessed whether any of the mitochondrial impairments observed with COPD relate to the exacerbated muscle atrophy in patients. In addressing this point, it is relevant that one prior study noted an increased release of cytochrome c from mitochondria isolated from COPD patient muscle (Puente-Maestu et al., 2009b). As noted above, cytochrome c release from mitochondria can occur in response to an event known as mitochondrial permeability transition (see beginning of this section) (Davidson et al., 2020), and is part of the pathway causing an increase in caspase 3 activation, with caspase 3 being an established mechanism causing muscle atrophy *via* actin cleavage (Du et al., 2004) and cleavage of negative regulators of the proteasome (Wang et al., 2010). Furthermore, mitochondrial permeability transition is also associated with an increase in mitochondrial ROS emission (Akopova et al., 2011), which is another established mechanism driving muscle atrophy (Hyatt et al., 2019). To directly test the potential for mitochondrial permeability transition to induce muscle atrophy, we recently showed that chemically inducing mitochondrial permeability transition in single muscle fibers causes atrophy that depends upon an increase in caspase 3 and mitochondrial ROS emission (Figure 3). Furthermore, muscle atrophy occurring in a single muscle fiber multi-day incubation model of denervation/disuse atrophy was prevented by inhibiting mitochondrial permeability transition (Burke et al., 2021). Thus, the previously observed increase in cytochrome c release from mitochondria isolated from COPD patient muscle (Puente-Maestu et al., 2009b) could reflect an increased occurrence of mitochondrial permeability transition, and for this reason it may be worthwhile to explore the therapeutic value of targeting of mitochondrial permeability transition as a means of attenuating exacerbated skeletal muscle atrophy in various conditions, including COPD.

## Significance of Denervation in Chronic Obstructive Pulmonary Disease Skeletal Muscle

The importance of cross-talk between the motoneuron and skeletal muscle fiber in regulating muscle phenotype is an area of continuing research. The pioneering work by the Eccles was amongst the first to investigate this area where they showed that surgically switching the nerve input between fast and slow muscles, such that fast muscle became innervated by slow motoneurons and slow muscle became innervated by fast motoneurons, yielded marked alterations in the contractile properties of the cross-innervated skeletal muscles (Buller et al., 1960). That this motoneuron-muscle fiber communication is bidirectional is supported by work showing that skeletal muscle-specific over-expression of peroxisome proliferator activated receptor -gamma coactivator 1 alpha (PGC-1α) leads to not only an increase in slow muscle fiber types but also an increased expression of the slow motoneuron-specific protein SVA2 in the motoneuron terminals (Chakkalakal et al., 2010), and this operates through PGC-1α-induced increase in the myokine neurturin in skeletal muscle (Correia et al., 2021). Although the importance of denervation in COPD muscle has not yet been widely studied, one of the first studies to hint at the involvement of denervation in COPD muscle phenotypes came from Gosker and colleagues where they reported the presence of very small muscle fibers (referred to as “minifibers” in this work) (Gosker et al., 2002), noting that the size and angular shape of these fibers, along with their frequent co-expression of multiple myosin heavy chain isoforms, are well-known attributes of neuromuscular diseases with motoneuron involvement such as amyotrophic lateral sclerosis (Baloh et al., 2007). In this respect, we recently examined COPD patient limb skeletal muscle to address the potential involvement of denervation and found numerous indications of ongoing denervation-reinnervation (fibers of the same type grouped together), along with evidence for persistent denervation of muscle fibers (accumulation of small fibers with angular shape, expression of the denervation-inducible glycoprotein neural cell adhesion molecule). Furthermore, patients with low muscle mass (based upon dual X-ray absorptiometry body composition) had higher fiber type grouping and a higher abundance of the very small muscle fibers, suggesting more denervation in the patients with low muscle mass. Strikingly, whereas patients who had maintained muscle mass exhibited a marked increase in expression of genes involved in re-establishing innervation following denervation (e.g., Agrin, muscle specific kinase (MuSK), and fibroblast growth factor binding protein 1 (FGFBP1)], patients with low muscle mass had a markedly blunted increase in these genes (Kapchinsky et al., 2018). We suggest that this implicates a failure of the muscle reinnervation response in the exacerbated accumulation of persistently denervated muscle fibers and resulting muscle atrophy in COPD. Consistent with an important role of denervation in COPD muscle, a recent study found significant correlations between circulating biomarkers of denervation (e.g., cleaved agrin fragment 22, brain derived neurotrophic factor) and the presence of muscle atrophy in male COPD patients (Karim et al., 2021). Based on the evidence so far, we suggest that further study of the involvement of, and mechanisms causing, denervation in COPD muscle is clearly warranted.

## Systemic Mechanisms of Skeletal Muscle Impairment in Chronic Obstructive Pulmonary Disease

At the systemic level, COPD has multifactorial consequences that include hypercapnia, hypoxemia (particularly during physical exertion), and inflammation, amongst others. These consequences are further compromised during acute exacerbations of disease severity, which can occur secondary to bacterial or viral infections (MacLeod et al., 2021). As a result of the high burden of smoking history that most often underlies COPD, and the frequent occurrence of major comorbidities in COPD patients (e.g., kidney disease, cardiovascular disease, etc.), there are many interacting mechanisms likely to contribute to the skeletal muscle impairment seen with COPD and it is not feasible to address every possibility in detail here. Amongst the most thoroughly addressed mechanisms considered to date are hypoxia, hypercapnia, inflammation and tobacco smoke, but other mechanisms are also emerging including chronic activation of the aryl hydrocarbon receptor, as will be reviewed below.

Skeletal muscle contractile function is affected by alterations in O_2_ supply through modulation of bioenergetic stress (Hepple, 2002), with hyperoxia being beneficial and hypoxia being detrimental, and the acute impairment of skeletal muscle function secondary to reduced muscle O_2_ delivery in COPD patients is well-known (Richardson et al., 1999; Broxterman et al., 2020). In addition to this acute effect of hypoxia, it is also thought that chronic exposure of skeletal muscle to a hypoxic *milieu* may contribute to chronic adverse muscle impact in COPD patients. Whereas there are many studies that have addressed the impact of chronic systemic hypoxia on skeletal muscle (Hoppeler and Vogt, 2001), most address more severe hypoxia than would typically be encountered in COPD patients. In addressing the impact of a relatively mild level of hypoxemia that has broader relevance to COPD patients, Debevec and colleagues (Debevec et al., 2018) recently examined changes in muscle mass by quantitative computed tomography and muscle fiber type in muscle biopsies in subjects undergoing 21 days of bedrest in normoxia vs. hypoxia (P_I_O_2_ = 90 mmHg, yielding a blood O_2_ saturation = 88 ± 2%). Their results showed that hypoxia further exacerbated both the decline of muscle mass and shift towards type IIx fibers seen with bed rest (Debevec et al., 2018), suggesting that, when present, systemic hypoxemia resulting from impaired pulmonary function with COPD is a potential contributor to the muscle impairment in patients, particularly when superimposed upon the very low level of habitual physical activity typical of patients.

The impact of hypercapnia on skeletal muscle alterations in COPD has also received some attention. As with studies on hypoxia, the levels of hypercapnia studied often exceed that seen in COPD patients, except perhaps under extreme conditions associated with acute exacerbations and/or in very severe patients needing lung transplant. Nonetheless, previous studies have shown that a PaCO_2_ level of approximately 75 mmHg is associated with muscle atrophy (Jaitovich et al., 2015; Ceco et al., 2020), and in C2C12 muscle cell culture yields an increase in mitochondrial respiratory capacity that appears to be due to a compensatory upregulation of mitochondria (Ceco et al., 2020). Thus, whilst hypercapnia may contribute to the exacerbated muscle atrophy observed in COPD, it is possible that it may have a beneficial compensatory impact in limiting the severity of muscle mitochondrial respiratory capacity (Balnis et al., 2020b). The mechanisms underlying hypercapnia-mediated muscle atrophy remain under study, but current evidence implicates activation of the energy sensor AMP Kinase (AMPK), particularly the AMPKα2 isoform, which in turn leads to increased expression of ubiquitin ligases such as MuRF1 (Jaitovich et al., 2015). Further to this, there is also recent evidence showing that COPD patients exhibiting hypcapnia had suppressed ribosomal gene expression, and mice exposed to chronically elevated CO_2_ levels had reduced ribosomal biogenesis and reduced protein synthesis based upon puromycin incorporation into skeletal muscle (Korponay et al., 2020). On this basis, it does appear that hypercapnia can be one of the systemic factors contributing to skeletal muscle atrophy in COPD through both suppressed protein synthesis and increased protein degradation.

Systemic inflammation is one of the best established features of COPD systemic pathophysiology and is particularly relevant during periods of acute exacerbations (MacLeod et al., 2021). Interestingly, inflammation is implicated in a wide variety of conditions involving muscle wasting, such as cancer cachexia and rheumatoid arthritis (Londhe & Guttridge, 2015; Webster et al., 2020), and has been frequently posited as a contributor to muscle atrophy in COPD (Maltais et al., 2014). For example, two recent studies have examined relationships between various circulating inflammatory cytokines and indices of muscle mass in COPD patients, where significant negative relationships between muscle mass and TNFα (Byun et al., 2017) and IL-6 (Lin et al., 2021) emerged following multivariate analysis. At a mechanistic level, a mouse model of chronic systemic inflammation consequent to knockout of IL10 exhibits various features of muscle impairment, including muscle atrophy, weakness, and neuromuscular junction alterations (Westbrook et al., 2020). Furthermore, increased NLRP3 inflammasome activity in muscle cell culture is associated with myotube atrophy (Liu et al., 2020), whereas NLRP3 inflammasome deficient mice have attenuated muscle loss with aging (Sayed et al., 2019). As such, chronic inflammation is likely to be an important contributor to muscle atrophy in COPD patients, and one that becomes even more important during acute exacerbations. It also important to consider that patients admitted to hospital during acute exacerbations are very physically inactive, which can be an additional factor contributing to muscle atrophy in COPD (Maltais et al., 2014).

**FIGURE 4:**
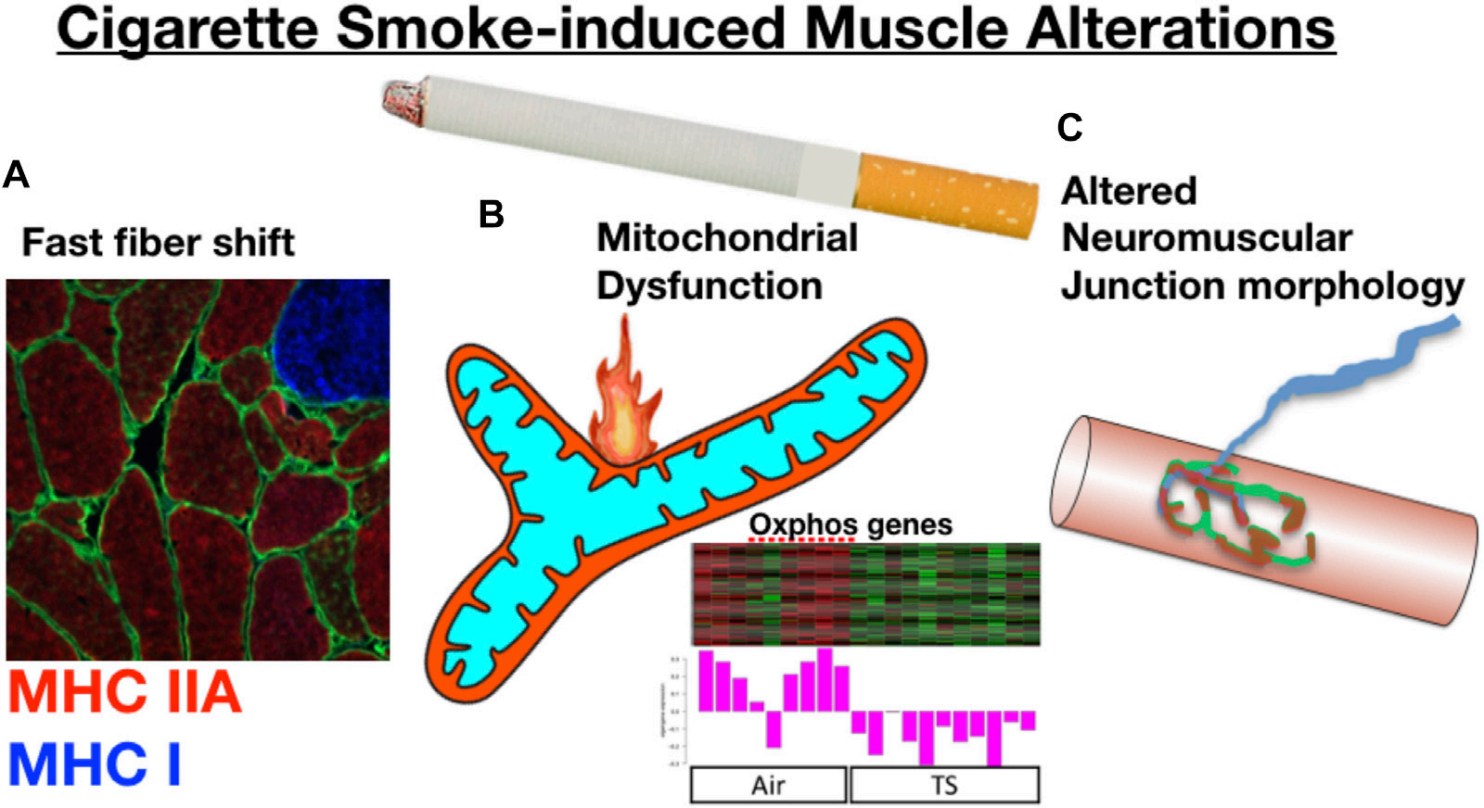
Prolonged cigarette smoking alone yields skeletal muscle changes that resonate with those seen in COPD patients. For example, long-term smokers without evidence of lung disease demonstrate **(A)** a fast fiber shift, and data from a smoking mouse model show that chronic cigarette smoke exposure leads to **(B)** reduced mitochondrial respiratory capacity that is associated with a down-regulation of mitochondrial genes, and **(C)** marked alterations to the neuromuscular junction. Oxphos gene panel modified from (Thome et al., 2022).

In addressing the specific role of chronic tobacco smoke (TS) exposure in the etiology of COPD muscle impairment, there is growing evidence that long-term cigarette smoking plays a key role in initiating the muscle impairments with COPD (as summarized in Figure 4). First, similar to what is reported in COPD patients (Gosker et al., 2007), Orlander and colleagues previously showed that skeletal muscle of smokers who had no evidence of lung disease demonstrated a pronounced fast fiber shift (Orlander et al., 1979), and this was true even when controlling for genetic predisposition for muscle fiber type (Larsson and Orlander, 1984). Smoking is also amongst the most consistent lifestyle factors associated with a greater degree of muscle atrophy with normal aging, independent of lung disease (Castillo et al., 2003; Szulc et al., 2004; Lee et al., 2007; van den Borst et al., 2011; Hashemi et al., 2016). Other groups have employed smoking mouse models, finding that chronic TS exposure in mice yields atrophy and fiber type shift (Gosker et al., 2009; Basic et al., 2012; Rinaldi et al., 2012; Caron et al., 2013), consistent with the observations in human smokers (Gosker et al., 2007; van den Borst et al., 2011). Smoking mouse studies have also identified a reduced skeletal muscle mitochondrial respiratory capacity (Fukuda et al., 2017; Perez-Rial et al., 2020; Thome et al., 2022), although this is not seen in all studies (Bowen et al., 2017; Decker et al., 2021). Furthermore, we recently showed that chronic TS exposure in mice causes morphological adaptations at the neuromuscular junction, which we propose plays a key role in the motor unit remodeling (fast fiber shift, type grouping), expression of histological markers of denervation (high non-specific esterase activity, high neural cell adhesion molecule [NCAM] expression), and transcriptional indicators of denervation in COPD patient muscle (Kapchinsky et al., 2018). In a follow-up study using a smoking mouse model, we showed that TS-induced neuromuscular junction alterations also involve the diaphragm (Thome et al., 2022), which likely plays a role in the diaphragm involvement seen in COPD patients (Ottenheijm et al., 2008). In addition to TS being implicated in the atrophy and fast fiber shift seen in COPD patient limb muscle, there is also evidence that chronic TS exposure causes increased muscle fatigue (Wust et al., 2008), which is another manifestation of skeletal muscle impact in COPD patients (Allaire et al., 2004; Bachasson et al., 2013). It is important to note that ascribing a role for chronic TS exposure in precipitating COPD muscle phenotypes does not in any way rule out the additional contributions of systemic COPD pathophysiology (e.g., hypoxia, hypercapnia, inflammation, etc.) discussed above. Notwithstanding this important distinction, it is also relevant to note that despite the abundance of evidence that chronic TS exposure contributes to the muscle impairment in COPD patients, the mechanisms underlying the impact of chronic TS exposure have, until recently, been largely unknown.

**FIGURE 5:**
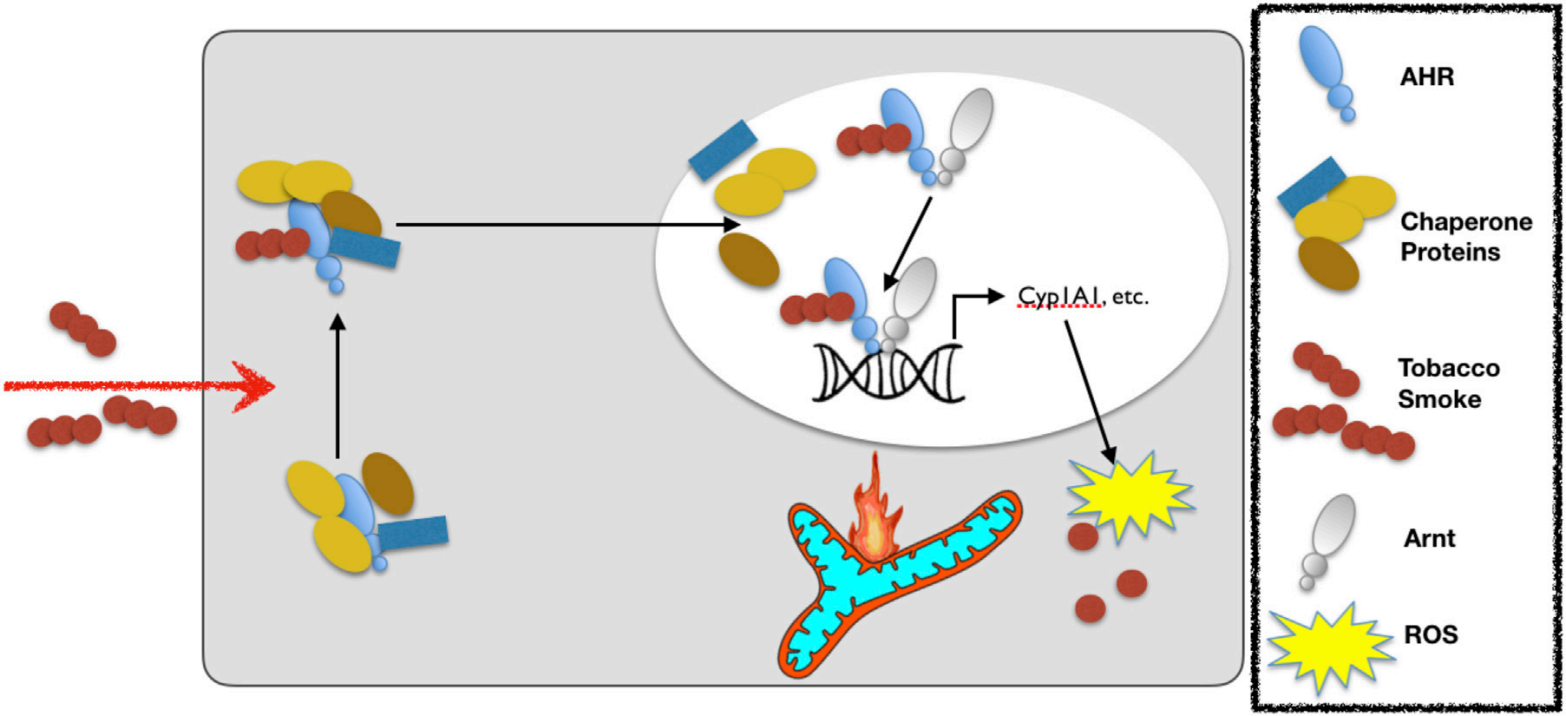
Exposure to cigarette smoke leads to activation of the aryl hydrocarbon receptor (AHR). Upon cigarette smoke constituent binding to the AHR, the AHR translocates to the nucleus and dissociates from its chaperones before binding to the aryl hydrocarbon receptor nuclear translocase (Arnt) and then binding with the so-called DNA response element (or xenobiotic response element) of target genes to up-regulate the expression of detoxifying cytochrome P450 enzymes (e.g., Cyp1A1). Notably, whereas acute activation of the AHR is adaptive, chronic activation leads to ROS-mediated stress and mitochondrial dysfunction that leads to toxicity. The legend defining each of the components in this schematic is in the box to the right.

**FIGURE 6:**
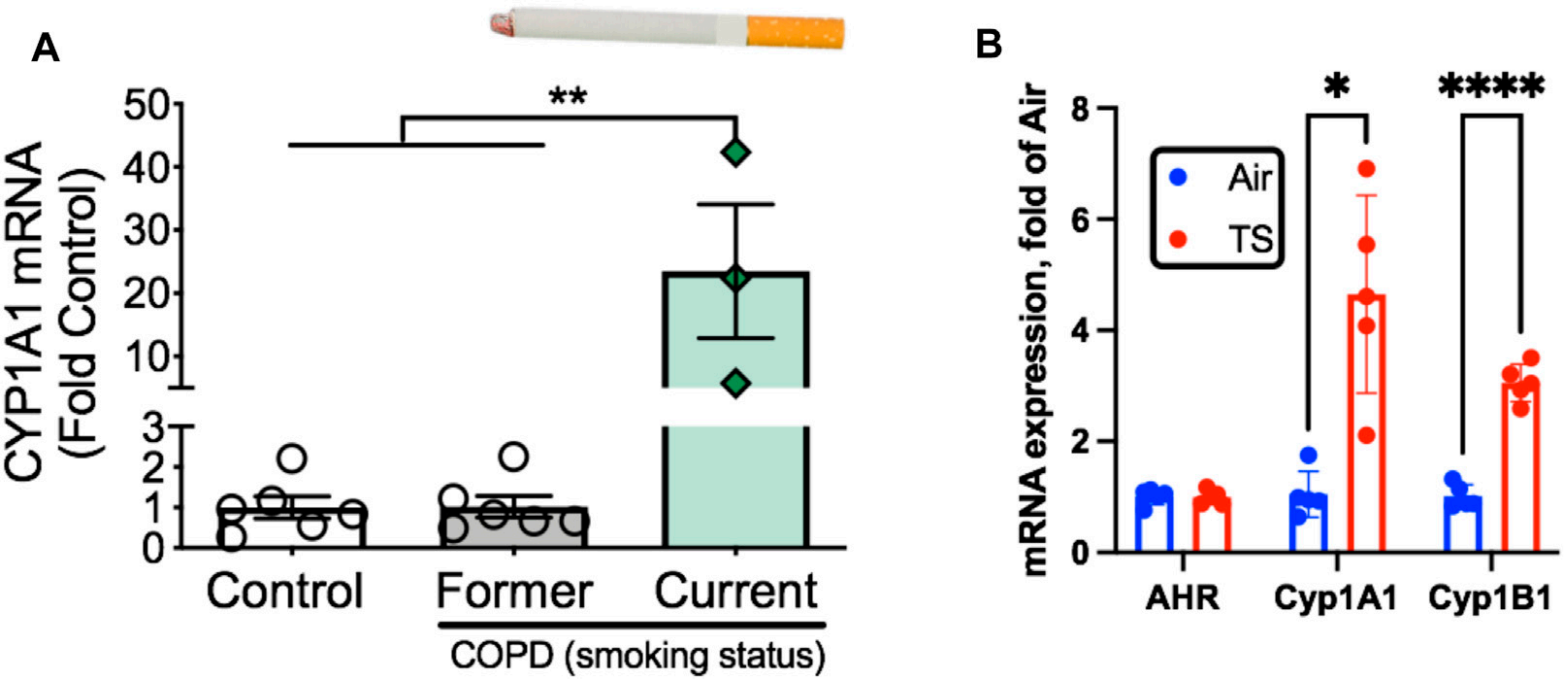
Tobacco smoke exposure activates AHR signaling in skeletal muscle. **(A)** AHR signaling (Cyp1A1 expression) is elevated exclusively in COPD patients who are current smokers but not those who were former smokers. **(B)** Acute tobacco smoke exposure (2 × 60 min exposures separated by 30 min) in mice leads to increases AHR signaling in skeletal muscle (Cyp1A1, Cyp1B1). Graphs are adapted from Thome et al., 2022.

**FIGURE 7:**
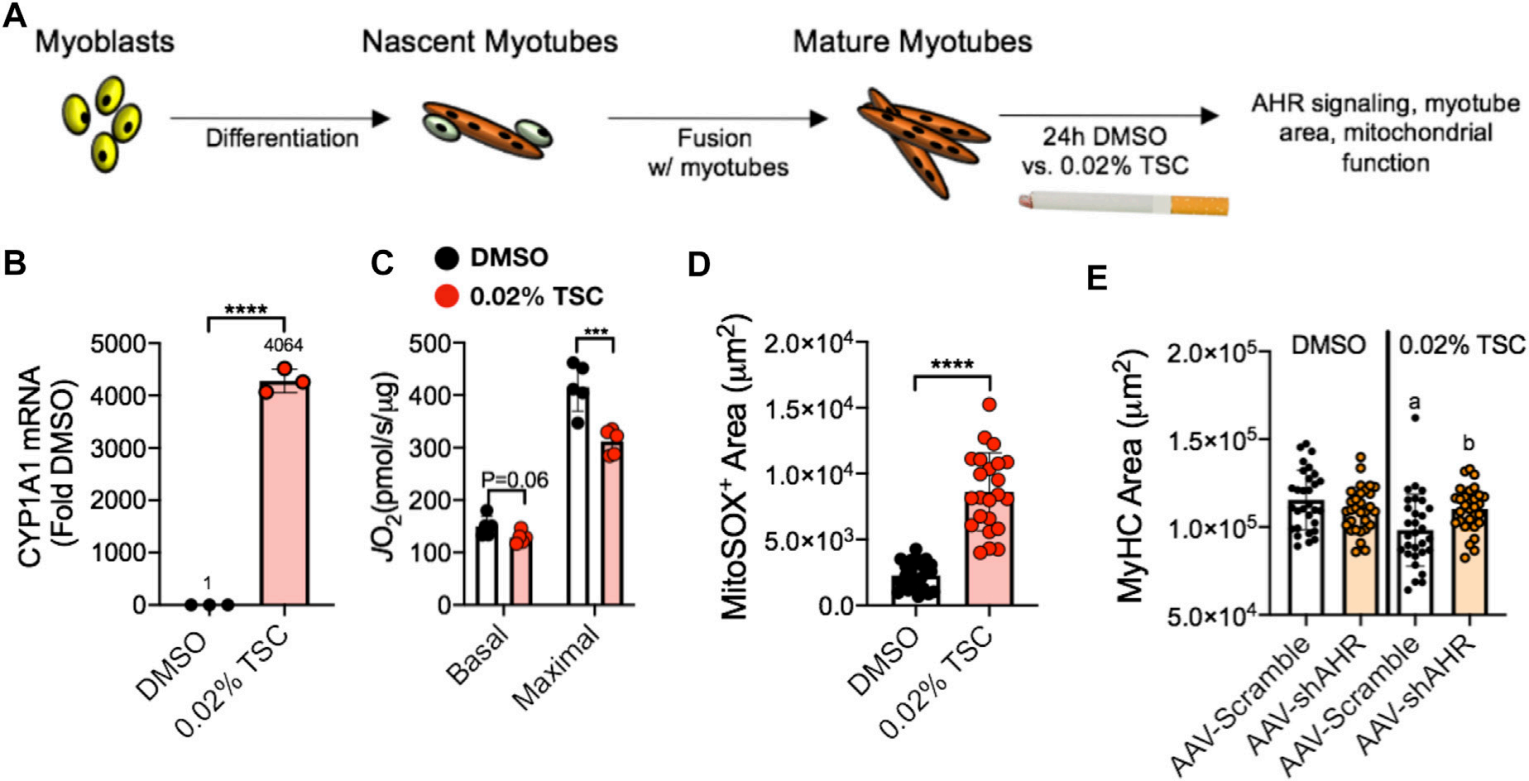
**(A)** Tobacco smoke condensate (TSC) exposure in C2C12 myotubes **(B)** activates AHR signaling (Cyp1A1), **(C)** reduces mitochondrial respiratory capacity (JO2), **(D)** increases mitochondrial ROS (detected using a MitoSox probe), and **(E)** leads to reduced myotube size that is blocked by shRNA targeting the AHR (shAHR) delivered by adeno-associated virus (AAV). Figure panels adapted from (Thome et al., 2022).

**FIGURE 8:**
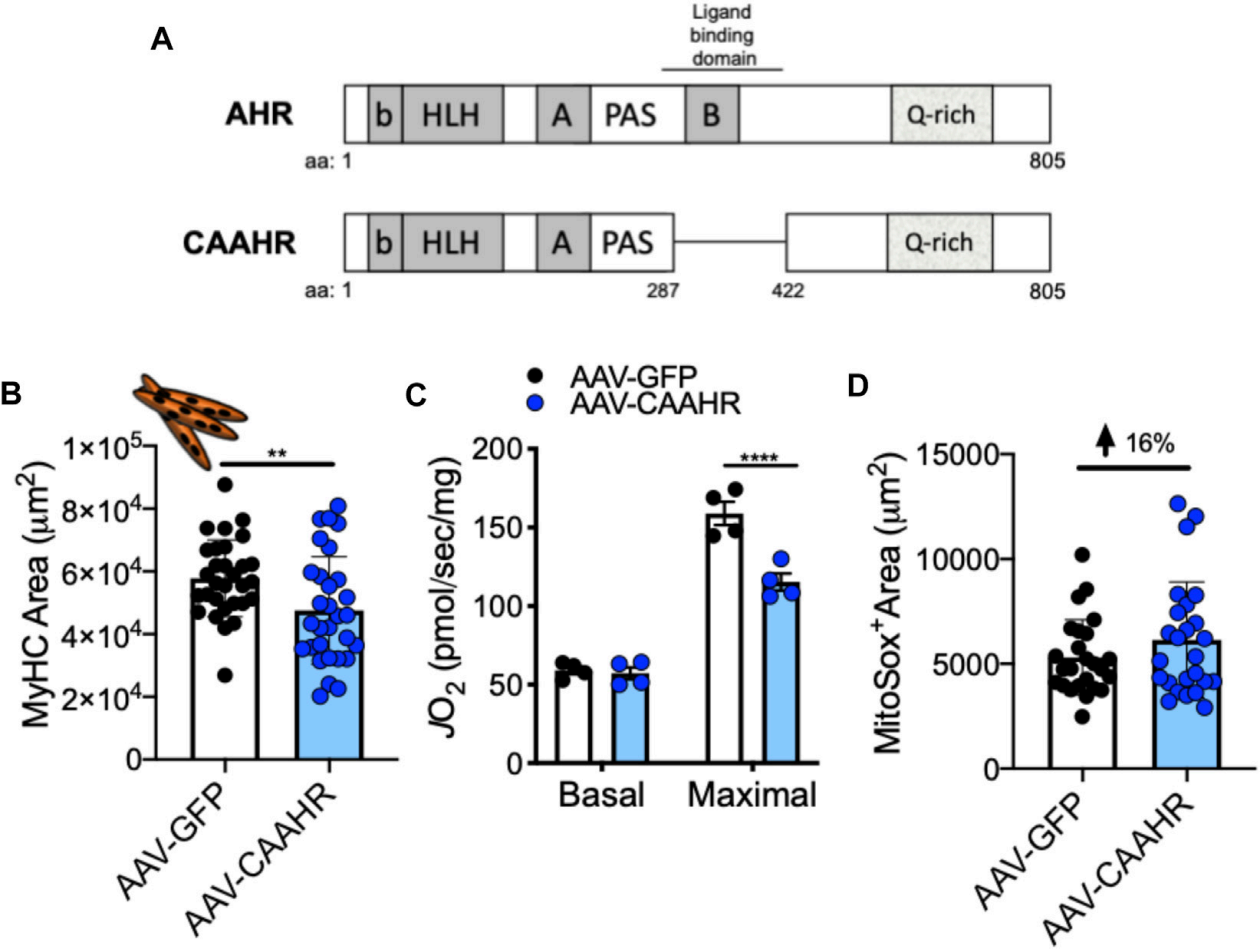
**(A)** Removal of a region containing the ligand binding domain from the AHR yields a mutant with constitutive AHR transcriptional activity (CAAHR). AAV-mediated transduction of the CAAHR phenocopies muscle and mitochondrial phenotypes produced by chronic tobacco smoke exposure, including **(B)** a reduction in myotube area, **(C)** reduced mitochondrial respiratory capacity (JO2), and **(D)** increased mitochondrial ROS emission (based upon a MitoSox probe). Graphs are adapted from (Thome et al., 2022).

**FIGURE 9:**
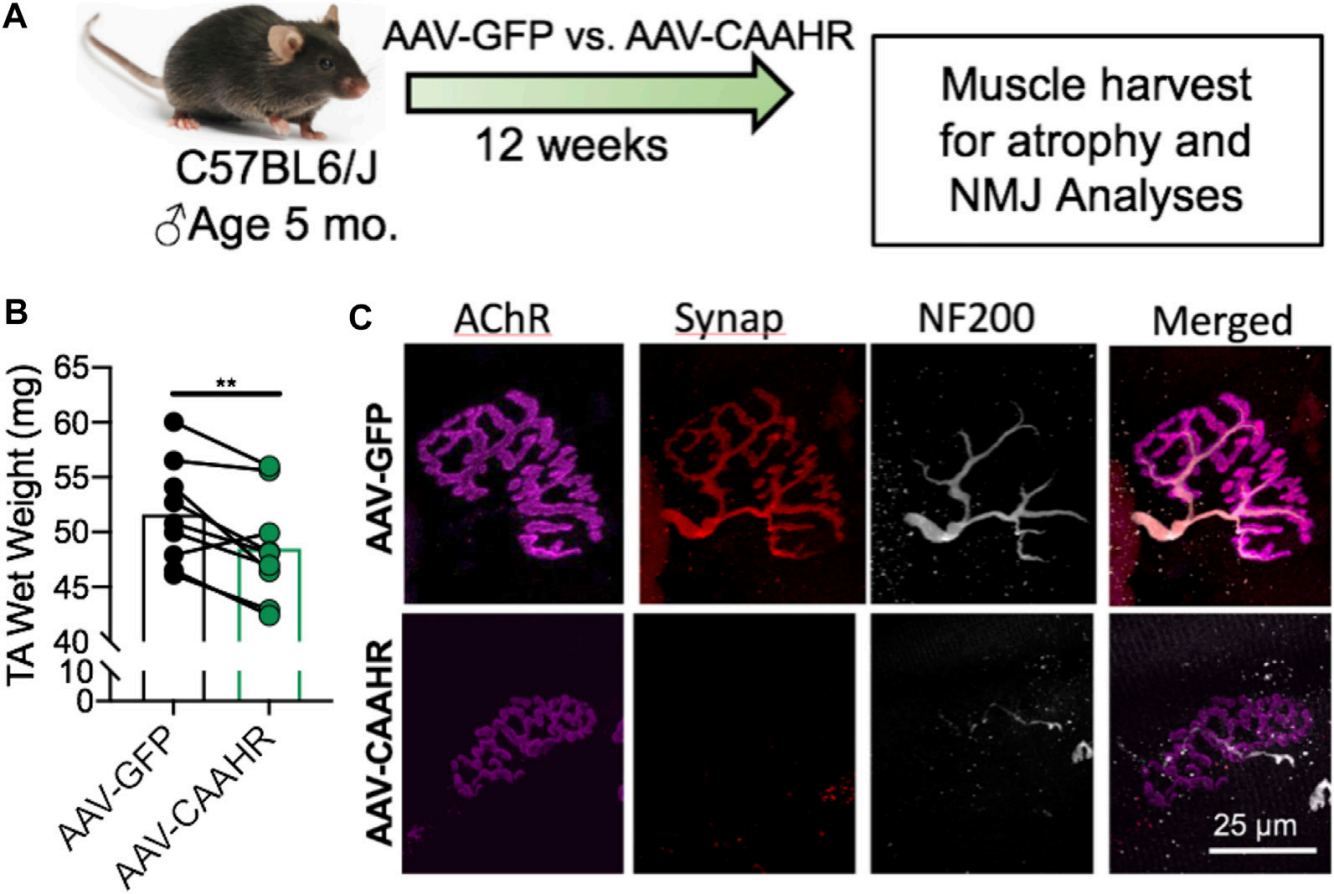
**(A)** Adeno-associated virus (AAV)-mediated delivery of a constitutively active mutant of the AHR (CAAHR) to skeletal muscle in mice yields **(B)** reduced muscle mass, and **(C)** alterations to the neuromuscular junction morphology that are strikingly similar to those produced by chronic exposure to tobacco smoke in mice. Graph and images are adapted from (Thome et al., 2022).

The aryl hydrocarbon receptor (AHR) is a ligand-activated transcription factor with a wide variety of endogenous and exogenous ligands that leads to increased transcription of detoxifying pathways, including cytochrome P450 enzymes such as Cyp1A1 (Nebert, 2017) (Figure 5, above). The AHR is perhaps best known for mediating much of the toxicity associated with dioxin poisoning (McIntosh et al., 2010), and it is noteworthy that mitochondrial dysfunction is implicated as a key mechanism responsible for the toxicity associated with chronic AHR activation (Senft et al., 2002; Forgacs et al., 2010; Zhou et al., 2017). Since TS activates the AHR (Kitamura and Kasai, 2007), and muscle impairment has been mentioned in some instances where chronic AHR activation occurs (Max and Silbergeld, 1987; Yi et al., 2014), we recently undertook studies to examine the role of the AHR in mediating muscle impairment with chronic TS exposure. Our initial experiments confirmed that AHR signaling (e.g., the cytochrome P450 enzymes Cyp1A1 and Cyp1B1) is elevated in the skeletal muscle of human smokers (Figure 6A) and in mice acutely exposed to TS (Figure 6B), and that chronic activation of the AHR by TS condensate in skeletal muscle cell culture (Figure 7A) elevates AHR signaling, impairs mitochondrial respiratory capacity, increases mitochondrial ROS emission, and causes an AHR-dependent myotube atrophy (Figures 7B–E). We then showed that knock-in of a constitutively active mutant of the AHR (demonstrates transcription factor activity without ligand) (Figure 8A) into skeletal muscle cells *in vitro* recapitulates the muscle atrophy (Figure 8B) and mitochondrial impairment seen with TS condensate (Figures 8C,D). Finally, in mice 12 weeks following intramuscular injection of this constitutively active AHR mutant using an AAV vector revealed muscle atrophy and altered neuromuscular junction morphology that was similar to what was seen with 16 weeks of chronic TS exposure in mice (Thome et al., 2022) (Figure 9). These findings suggest that TS-induced chronic activation of the AHR is likely to be an important contributor to the mitochondrial impairment, muscle atrophy and denervation phenotypes seen in COPD patients, and clearly warrants additional study.

Interestingly, the role of chronic AHR activity in mediating adverse muscle impact in COPD may not be exclusive to the effects of TS on AHR activation. As noted above, several disease comorbidities are seen in COPD patients. Amongst these comorbid diseases is chronic kidney disease, where recent data indicates approximately 7% of COPD patients also have chronic kidney disease (Trudzinski et al., 2019), with many more patients expected to have subclinical kidney disease (i.e., renal impairment that is below the clinical threshold for disease diagnosis). Notably, the impaired ability of the kidney to filter out various toxic metabolites (so-called uremic toxins) is thought to be an important contributor to muscle atrophy (Schardong et al., 2018) and mitochondrial impairment (Thome et al., 2019) in patients with chronic kidney disease, and subclinical kidney disease has also been implicated as a significant exacerbating factor for muscle atrophy in the elderly (Lustgarten and Fielding, 2017). Interestingly, blood serum from patients with chronic kidney disease activates AHR signaling (Dou et al., 2018), consistent with evidence that several of the uremic toxins activate the AHR, including the tryptophan metabolite kynurenine (Mezrich et al., 2010). In this latter respect, a recent study revealed an elevated circulating kynurenine level in COPD patients (Gosker et al., 2019), suggesting that kynurenine-mediated AHR activation could contribute to COPD muscle impairment, in addition to the aforementioned impact of TS-mediated AHR activation. As such, the available evidence provides a strong rationale for further studies to address the role played by chronic AHR activation in mediating the toxic muscle effects of multiple aspects of the COPD systemic *milieu*.

## Chronic Obstructive Pulmonary Disease and Exacerbated Muscle Aging

**FIGURE 10:**
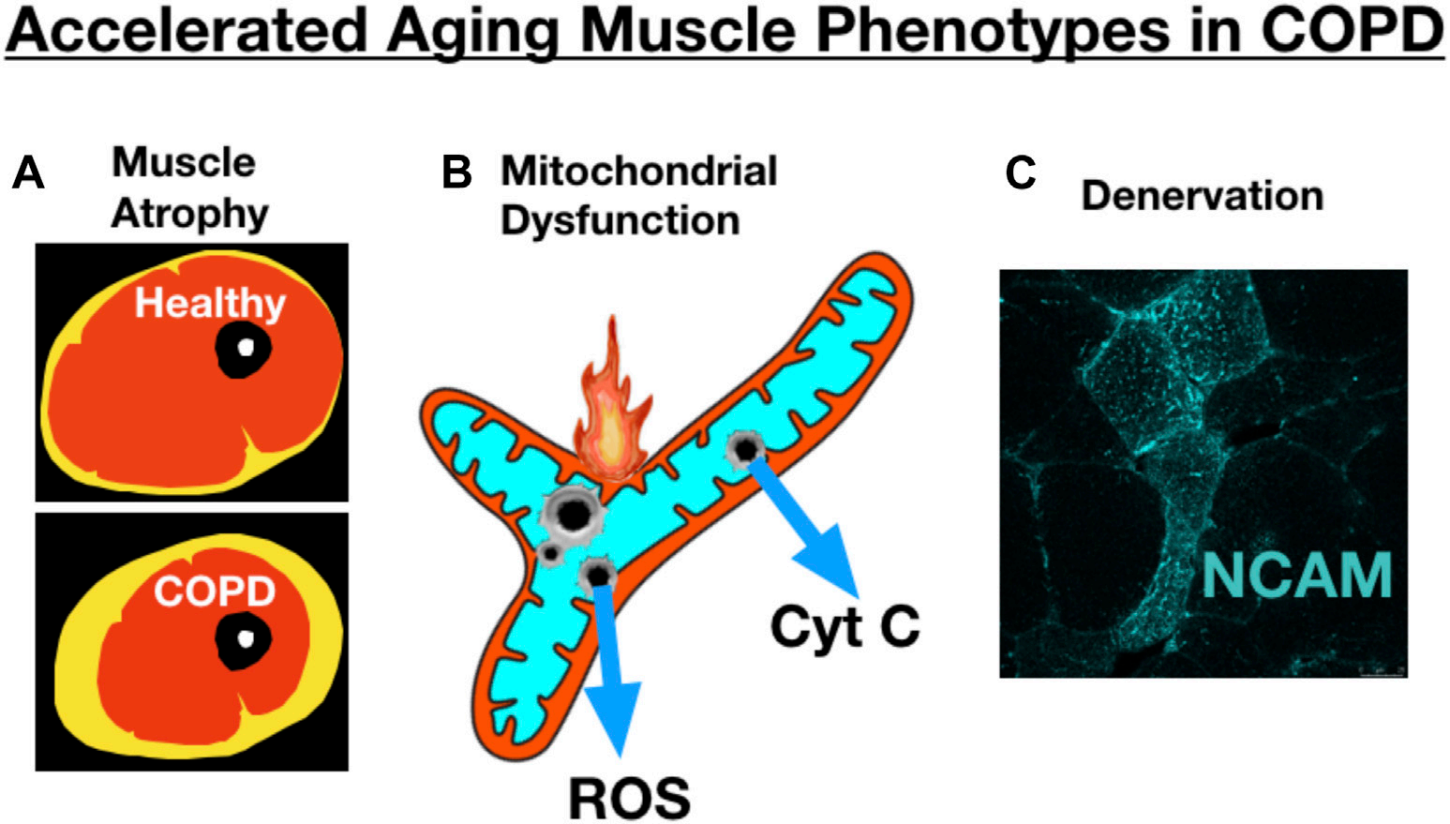
COPD skeletal muscle exhibits several traits that resemble accelerated aging, including exacerbation of **(A)** atrophy (schematic image of an MRI through the thigh showing skeletal muscle, adipose tissue, bone), **(B)** mitochondrial dysfunction, and **(C)** accumulation of muscle fibers expressing markers of denervation (e.g., neural cell adhesion molecule [NCAM] expression). Image showing NCAM labeling adapted from (Kapchinsky et al., 2018).

Several studies have addressed whether COPD may accelerate fundamental aging biology in an attempt to understand whether this may underlie some of the adverse muscle impact in COPD. In this respect, it is relevant to point out some key similarities (Figure 10) and differences between aging and COPD muscle phenotypes. Amongst the most obvious similarities is muscle atrophy, where, as noted from the beginning of this review, COPD patients exhibit more atrophy than is typical of age-matched healthy subjects. Similarly, reduced mitochondrial respiratory capacity, increased mitochondrial ROS and increased incidence of mitochondrial permeability transition are all properties of aging skeletal muscle mitochondria (Gouspillou et al., 2014; Spendiff et al., 2016; Sonjak et al., 2019b) that appear to be further impaired in skeletal muscle of COPD patients (Picard et al., 2008; Puente-Maestu et al., 2009a; Puente-Maestu et al., 2009b). Finally, the well-known accumulation of muscle fibers exhibiting markers of persistent denervation and fiber type grouping that reflect recurring cycles of denervation-reinnervation with aging (Messi et al., 2016; Sonjak et al., 2019a) are further potentiated in skeletal muscle of COPD patients (Kapchinsky et al., 2018). On the other hand, COPD patients frequently exhibit a pronounced shift towards an increased abundance of fast twitch muscle fibers (Gosker et al., 2007; Kapchinsky et al., 2018), and this is not typical of normal aging where the primary impact is an increased abundance of fibers expressing multiple myosin heavy chains (co-expressing fibers) with little change in the overall proportion of slow vs. fast fibers (Andersen, 2003; Kelly et al., 2018; Sonjak et al., 2019a). Thus, whereas there is some overlap in muscle phenotypes between COPD and aging, consistent with the possibility of COPD causing an exacerbation of fundamental aging biology, there may also be some mechanisms that distinguish COPD from exacerbated aging at least at the gross muscle phenotypic level.

One of the challenges in determining whether COPD exacerbates fundamental aging biology is selecting appropriate biomarkers to represent the severity of aging muscle impact. For example, amongst the most commonly used biomarkers of cellular senescence are the cyclin-dependent kinase inhibitor p16, senescence associated β-Galactoside (SA β-Gal), and the histone H2A variant gamma-H2AX (γH2AX). These senescence markers were recently evaluated in the context of aging human skeletal muscle using immunolabeling of muscle cross-sections from young (18–35 years) vs. old (>70 years) subjects. The authors noted that despite using numerous commercially available antibodies for each senescence marker, γH2AX was the only one detectable in human muscle, and strikingly it did not evidence an increase with aging and instead was elevated exclusively in myonuclei of obese vs. lean subjects (Dungan et al., 2020). Keeping in mind this important caveat suggesting classical senescence markers may not be informative in gauging the impact of aging on skeletal muscle, another recent study employing Western blot for protein quantification reported reduced γH2AX protein levels in muscle of COPD patients (Lakhdar et al., 2018). Although further analyses may alter our understanding, based upon current knowledge, adverse skeletal muscle impact in COPD seems unlikely to be consequent to exacerbated accumulation of senescent cells.

Another biomarker of aging that has received some attention in the context of COPD skeletal muscle impairment is the anti-aging hormone α-klotho (hereafter referred to as klotho). Klotho serves as a co-receptor for fibroblast growth factor 23 to activate a variety of fibroblast growth factor receptors throughout the body (Cheikhi et al., 2019), and is necessary for normal mitochondrial function in a variety of tissues (Chen et al., 2020; Lee et al., 2021), including skeletal muscle (Sahu et al., 2018). Various studies have implicated klotho in normal aging of skeletal muscle, with studies showing that reduced circulating levels of klotho in the blood with aging correlate with poor grip strength (Semba et al., 2012), reduced knee extensor strength (Semba et al., 2016), and impaired muscle regeneration (Sahu et al., 2018), amongst other age-related issues. Further to this, klotho knockout mice exhibit features of premature aging (Kuro-o et al., 1997), including a reduction in muscle strength and increased fatigability (Phelps et al., 2013). Interestingly, there is some evidence for reduced serum klotho levels in COPD that appears to be driven by smoking (Kureya et al., 2016; Patel et al., 2016), and serum but not muscle levels of klotho appear to be predictive of quadriceps muscle strength (Patel et al., 2016). On the other hand, another study found no association of plasma klotho levels with clinical parameters, including strength, in COPD patients undergoing pulmonary rehabilitation (Pako et al., 2017). Thus, based on current knowledge, the importance of klotho in the muscle impairment in COPD remains an interesting idea, but with mixed evidence.

## Summary and Conclusion

Skeletal muscle of COPD patients is characterized by an exacerbated degree of atrophy relative to age-matched healthy subjects. They also exhibit other manifestations of skeletal muscle impairment, including exacerbated fatigue, weakness, a fast fiber shift, mitochondrial dysfunction, and an accumulation of denervated muscle fibers that is associated with a failed reinnervation transcriptional profile. Notably, COPD patient skeletal muscle exhibits evidence of an increased frequency of mitochondrial permeability transition events, where recent evidence identifies mitochondrial permeability transition as a mechanism of muscle atrophy operating through mitochondrial ROS and caspase 3. There are likely multiple contributing factors underlying the skeletal muscle alterations in COPD, including chronic TS exposure, systemic hypoxia, systemic hypercapnia, and inflammation. Notably, smoking mouse models show that chronic TS exposure without overt lung disease can cause muscle atrophy, mitochondrial impairment, and neuromuscular junction alterations, underscoring the likely role of long-term TS exposure in initiating the muscle impairment in COPD. Furthermore, recent evidence suggests that chronic activation of the AHR in skeletal muscle is likely to play an important role in driving these TS-induced muscle alterations as constitutive activation of the AHR without TS exposure recapitulates many of the same muscle phenotypes induced by chronic TS exposure. In addition to TS-mediated AHR activation, the higher circulating level of the tryptophan metabolite kynurenine seen in COPD patients, which is likely a consequence of impaired renal function, can also activate the AHR. This suggests multiple pathological insults involved in COPD may converge upon the AHR in causing skeletal muscle toxicity. Although an exacerbated accumulation of senescent cells in COPD muscle seems unlikely to underlie the exacerbation of aging muscle phenotypes (atrophy, mitochondrial dysfunction, denervation), reduced circulating levels of the longevity-promoting klotho protein have been reported in COPD and some evidence suggests this may contribute to the adverse muscle phenotypes. In conclusion, COPD patient skeletal muscle alterations are likely to be driven by a combination of factors, some of which precede COPD disease diagnosis (e.g., TS-induced muscle alterations), and others that are part of the COPD patient’s systemic milieu (e.g., hypoxemia, hypercapnia, inflammation) and disease comorbidities (e.g., renal impairment). Based upon emerging evidence, preclinical models examining the potential of approaches targeting the mitochondrion (e.g., to inhibit mitochondrial permeability transition) and the aryl hydrocarbon receptor may be valuable in seeking new targeted therapies for treating and preventing adverse skeletal muscle impact in COPD patients.

## References

[B1] Akopova O. V. Kolchynskayia L. Y. Nosar' V. Y. Smyrnov A. N. Malisheva M. K. Man'kovskaia Y. N. (2011). The Effect of Permeability Transition Pore Opening on Reactive Oxygen Species Production in Rat Brain Mitochondria. Ukr. Biokhim Zh (1999) 83, 46–55. 22364018

[B2] Allaire J. Maltais F. Doyon J. F. Noel M. LeBlanc P. Carrier G. (2004). Peripheral Muscle Endurance and the Oxidative Profile of the Quadriceps in Patients with COPD. Thorax 59, 673–678. 10.1136/thx.2003.020636 15282387 PMC1747097

[B3] Alway S. E. Mohamed J. S. Myers M. J. (2017). Mitochondria Initiate and Regulate Sarcopenia. Exerc Sport Sci. Rev. 45, 58–69. 10.1249/jes.0000000000000101 28098577 PMC5357179

[B4] Anagnostou M. E. Hepple R. T. (2020). Mitochondrial Mechanisms of Neuromuscular Junction Degeneration with Aging. Cells 9 (1), 197. 10.3390/cells9010197 PMC7016881 31941062

[B5] Andersen J. L. (2003). Muscle Fibre Type Adaptation in the Elderly Human Muscle. Scand. J. Med. Sci. Sports. 13, 40–47. 10.1034/j.1600-0838.2003.00299.x 12535316

[B6] Anderson L. J. Liu H. Garcia J. M. (2017). Sex Differences in Muscle Wasting. Adv. Exp. Med. Biol. 1043, 153–197. 10.1007/978-3-319-70178-3_9 29224095

[B7] Attaway A. H. Welch N. Hatipoğlu U. Zein J. G. Dasarathy S. (2021). Muscle Loss Contributes to Higher Morbidity and Mortality in COPD: An Analysis of National Trends. Respirology 26, 62–71. 10.1111/resp.13877 32542761

[B8] Bachasson D. Wuyam B. Pepin J.-L. Tamisier R. Levy P. Verges S. (2013). Quadriceps and Respiratory Muscle Fatigue Following High-Intensity Cycling in COPD Patients. PLoS One 8, e83432. 10.1371/journal.pone.0083432 24324843 PMC3855800

[B9] Balnis J. Drake L. A. Vincent C. E. Korponay T. C. Singer D. V. Lacomis D. (2021). SDH Subunit C Regulates Muscle Oxygen Consumption and Fatigability in an Animal Model of Pulmonary Emphysema. Am. J. Respir. Cell. Mol. Biol. 65, 259–271. 10.1165/rcmb.2020-0551oc 33909984 PMC8485989

[B10] Balnis J. Korponay T. C. Vincent C. E. Singer D. V. Adam A. P. Lacomis D. (2020a). IL-13-driven Pulmonary Emphysema Leads to Skeletal Muscle Dysfunction Attenuated by Endurance Exercise. J. Appl. Physiolo. 128, 134–148. 10.1152/japplphysiol.00627.2019 PMC7054638 31774358

[B11] Balnis J. Lee C. G. Elias J. A. Jaitovich A. (2020b). Hypercapnia-Driven Skeletal Muscle Dysfunction in an Animal Model of Pulmonary Emphysema Suggests a Complex Phenotype. Front. Physiol. 11, 600290. 10.3389/fphys.2020.600290 33192616 PMC7658396

[B12] Baloh R. H. Rakowicz W. Gardner R. Pestronk A. (2007). Frequent Atrophic Groups with Mixed-type Myofibers Is Distinctive to Motor Neuron Syndromes. Muscle Nerve 36, 107–110. 10.1002/mus.20755 17299742

[B13] Basic V. T. Tadele E. Elmabsout A. A. Yao H. Rahman I. Sirsjö A. (2012). Exposure to cigarette smoke induces overexpression of von Hippel-Lindau tumor suppressor in mouse skeletal muscle. Am. J. Physiol.-Lung Cell. Mol. Physiolo. 303, L519–L527. 10.1152/ajplung.00007.2012 PMC3468481 22842216

[B14] Bauer T. M. Murphy E. (2020). Role of Mitochondrial Calcium and the Permeability Transition Pore in Regulating Cell Death. Circ. Res. 126, 280–293. 10.1161/circresaha.119.316306 31944918 PMC8317591

[B15] Benz E. Trajanoska K. Schoufour J. D. Lahousse L. de Roos E. W. Terzikhan N. (2021). Sarcopenia in Older People with Chronic Airway Diseases: the Rotterdam Study. ERJ Open Res. 7 (1), 00522-2020. 10.1183/23120541.00522-2020 33718493 PMC7938051

[B16] Bowen T. S. Aakerøy L. Eisenkolb S. Kunth P. Bakkerud F. Wohlwend M. (2017). Exercise Training Reverses Extrapulmonary Impairments in Smoke-Exposed Mice. Med. Sci. Sports. Exerc. 49, 879–887. 10.1249/mss.0000000000001195 28009790

[B17] Broxterman R. M. Hoff J. Wagner P. D. Richardson R. S. (2020). Determinants of the Diminished Exercise Capacity in Patients with Chronic Obstructive Pulmonary Disease: Looking beyond the Lungs. J. Physiol. 598, 599–610. 10.1113/jp279135 31856306 PMC6995414

[B18] Bua E. Johnson J. Herbst A. Delong B. McKenzie D. Salamat S. (2006). Mitochondrial DNA-Deletion Mutations Accumulate Intracellularly to Detrimental Levels in Aged Human Skeletal Muscle Fibers. Am. J. Hum. Genet. 79, 469–480. 10.1086/507132 16909385 PMC1559550

[B19] Buller A. J. Eccles J. C. Eccles R. M. (1960). Interactions between Motoneurones and Muscles in Respect of the Characteristic Speeds of Their Responses. J. Physiol. 150, 417–439. 10.1113/jphysiol.1960.sp006395 13805874 PMC1363172

[B20] Burke S. K. Solania A. Wolan D. W. Cohen M. S. Ryan T. E. Hepple R. T. (2021). Mitochondrial Permeability Transition Causes Mitochondrial Reactive Oxygen Species- and Caspase 3-Dependent Atrophy of Single Adult Mouse Skeletal Muscle Fibers. Cells 10, 2586. 10.3390/cells10102586 34685566 PMC8534155

[B21] Byun M. K. Cho E. N. Chang J. Ahn C. M. Kim H. J. (2017). Sarcopenia Correlates with Systemic Inflammation in COPD. Int. J. Chron. Obstruct. Pulmon. Dis. 12, 669–675. 10.2147/copd.s130790 28255238 PMC5325093

[B22] Caron M.-A. Morissette M. C. Thériault M.-E. Nikota J. K. Stämpfli M. R. Debigaré R. (2013). Alterations in Skeletal Muscle Cell Homeostasis in a Mouse Model of Cigarette Smoke Exposure. PLoS One 8, e66433. 10.1371/journal.pone.0066433 23799102 PMC3682961

[B23] Castillo E. M. Goodman-Gruen D. Kritz-Silverstein D. Morton D. J. Wingard D. L. Barrett-Connor E. (2003). Sarcopenia in Elderly Men and Women. Am. J. Prev. Med. 25, 226–231. 10.1016/s0749-3797(03)00197-1 14507529

[B24] Ceco E. Celli D. Weinberg S. Shigemura M. Welch L. C. Volpe L. (2020). Elevated CO2 Levels Delay Skeletal Muscle Repair by Increasing Fatty Acid Oxidation. Front. Physiol. 11, 630910. 10.3389/fphys.2020.630910 33551852 PMC7859333

[B25] Chakkalakal J. V. Nishimune H. Ruas J. L. Spiegelman B. M. Sanes J. R. (2010). Retrograde Influence of Muscle Fibers on Their Innervation Revealed by a Novel Marker for Slow Motoneurons. Development 137, 3489–3499. 10.1242/dev.053348 20843861 PMC2947760

[B26] Cheikhi A. Barchowsky A. Sahu A. Shinde S. N. Pius A. Clemens Z. J. (2019). Klotho: An Elephant in Aging Research. J. Gerontol. A Biol. Sci. Med. Sci. 74 (7), 1031–1042. 10.1093/gerona/glz061 30843026 PMC7330474

[B27] Chen Z. Zhou Q. Liu C. Zeng Y. Yuan S. (2020). Klotho Deficiency Aggravates Diabetes-Induced Podocyte Injury Due to DNA Damage Caused by Mitochondrial Dysfunction. Int. J. Med. Sci. 17, 2763–2772. 10.7150/ijms.49690 33162804 PMC7645346

[B28] Corlateanu A. Covantev S. Mathioudakis A. G. Botnaru V. Siafakas N. (2016). Prevalence and Burden of Comorbidities in Chronic Obstructive Pulmonary Disease. Respir. Investig. 54, 387–396. 10.1016/j.resinv.2016.07.001 27886849

[B29] Correia J. C. Kelahmetoglu Y. Jannig P. R. Schweingruber C. Shvaikovskaya D. Zhengye L. (2021). Muscle-secreted Neurturin Couples Myofiber Oxidative Metabolism and Slow Motor Neuron Identity. Cell. Metab. 33, 2215–2230. e2218. 10.1016/j.cmet.2021.09.003 34592133

[B30] D'Agostino B. Polverino M. Cirino G. Lombardi A. Grassi B. Sullo N. (2010). Exercise Capacity and Cytochrome Oxidase Activity in Muscle Mitochondria of COPD Patients. Respir. Med. 104, 83–90. 10.1016/j.rmed.2009.07.016 19716278

[B31] Davidson S. M. Adameová A. Barile L. Cabrera‐Fuentes H. A. Lazou A. Pagliaro P. (2020). Mitochondrial and Mitochondrial‐independent Pathways of Myocardial Cell Death during Ischaemia and Reperfusion Injury. J. Cell. Mol. Med. 24, 3795–3806. 10.1111/jcmm.15127 32155321 PMC7171390

[B32] Debevec T. Ganse B. Mittag U. Eiken O. Mekjavic I. B. Rittweger J. (2018). Hypoxia Aggravates Inactivity-Related Muscle Wasting. Front. Physiol. 9, 494. 10.3389/fphys.2018.00494 29867545 PMC5962751

[B33] Decker S. T. Kwon O.-S. Zhao J. Hoidal J. R. Heuckstadt T. Richardson R. S. (2021). Skeletal Muscle Mitochondrial Adaptations Induced by Long-Term Cigarette Smoke Exposure. Am. J. Physiology-Endocrinology Metabo. 321, E80–E89. 10.1152/ajpendo.00544.2020 PMC8321829 34121449

[B34] Dou L. Poitevin S. Sallée M. Addi T. Gondouin B. McKay N. (2018). Aryl Hydrocarbon Receptor Is Activated in Patients and Mice with Chronic Kidney Disease. Kidney Int. 93, 986–999. 10.1016/j.kint.2017.11.010 29395338

[B35] Du J. Wang X. Miereles C. Bailey J. L. Debigare R. Zheng B. (2004). Activation of Caspase-3 Is an Initial Step Triggering Accelerated Muscle Proteolysis in Catabolic Conditions. J. Clin. Invest. 113, 115–123. 10.1172/jci18330 14702115 PMC300763

[B36] Dungan C. M. Peck B. D. Walton R. G. Huang Z. Bamman M. M. Kern P. A. (2020). *In Vivo* analysis of γH2AX+ Cells in Skeletal Muscle from Aged and Obese Humans. FASEB J. 34, 7018–7035. 10.1096/fj.202000111rr 32246795 PMC7243467

[B37] Forgacs A. L. Burgoon L. D. Lynn S. G. LaPres J. J. Zacharewski T. (2010). Effects of TCDD on the Expression of Nuclear Encoded Mitochondrial Genes. Toxicol. Appl. Pharmacol. 246, 58–65. 10.1016/j.taap.2010.04.006 20399798 PMC4030424

[B38] Fukuda T. Bouchi R. Takeuchi T. Nakano Y. Murakami M. Minami I. (2017). Association of Diabetic Retinopathy with Both Sarcopenia and Muscle Quality in Patients with Type 2 Diabetes: a Cross-Sectional Study. BMJ Open Diab Res. Care 5, e000404. 10.1136/bmjdrc-2017-000404 PMC5530250 28761661

[B39] Gosker H. R. Clarke G. de Theije C. C. Cryan J. F. Schols A. M. W. J. (2019). Impaired Skeletal Muscle Kynurenine Metabolism in Patients with Chronic Obstructive Pulmonary Disease. J. Clin. Med. 8 (7), 915. 10.3390/jcm8070915 PMC6678819 31247950

[B40] Gosker H. R. Engelen M. P. van Mameren H. van Dijk P. J. van der Vusse G. J. Wouters E. F. (2002). Muscle Fiber Type IIX Atrophy Is Involved in the Loss of Fat-free Mass in Chronic Obstructive Pulmonary Disease. Am. J. Clin. Nutr. 76, 113–119. 10.1093/ajcn/76.1.113 12081824

[B41] Gosker H. R. Langen R. C. J. Bracke K. R. Joos G. F. Brusselle G. G. Steele C. (2009). Extrapulmonary Manifestations of Chronic Obstructive Pulmonary Disease in a Mouse Model of Chronic Cigarette Smoke Exposure. Am. J. Respir. Cell. Mol. Biol. 40, 710–716. 10.1165/rcmb.2008-0312oc 18988919

[B42] Gosker H. R. Zeegers M. P. Wouters E. F. M. Schols A. M. W. J. (2007). Muscle Fibre Type Shifting in the Vastus Lateralis of Patients with COPD Is Associated with Disease Severity: a Systematic Review and Meta-Analysis. Thorax 62, 944–949. 10.1136/thx.2007.078980 17526675 PMC2117111

[B43] Gouspillou G. Sgarioto N. Kapchinsky S. Purves‐Smith F. Norris B. Pion C. H. (2014). Increased Sensitivity to Mitochondrial Permeability Transition and Myonuclear Translocation of Endonuclease G in Atrophied Muscle of Physically Active Older Humans. FASEB J. 28, 1621–1633. 10.1096/fj.13-242750 24371120

[B44] Hashemi R. Shafiee G. Motlagh A. D. Pasalar P. Esmailzadeh A. Siassi F. (2016). Sarcopenia and its Associated Factors in Iranian Older Individuals: Results of SARIR Study. Archives Gerontology Geriatrics 66, 18–22. 10.1016/j.archger.2016.04.016 27176487

[B45] Hepple R. T. (2016). Impact of Aging on Mitochondrial Function in Cardiac and Skeletal Muscle. Free Radic. Biol. Med. 98, 177–186. 10.1016/j.freeradbiomed.2016.03.017 27033952

[B46] Hepple R. T. (2002). The Role of O2 Supply in Muscle Fatigue. Can. J. Appl. Physiol. 27, 56–69. 10.1139/h02-004 11880691

[B47] Hood D. A. Memme J. M. Oliveira A. N. Triolo M. (2019). Maintenance of Skeletal Muscle Mitochondria in Health, Exercise, and Aging. Annu. Rev. Physiol. 81, 19–41. 10.1146/annurev-physiol-020518-114310 30216742

[B48] Hoppeler H. Vogt M. (2001). Muscle Tissue Adaptations to Hypoxia. J. Exp. Biol. 204, 3133–3139. 10.1242/jeb.204.18.3133 11581327

[B49] Hyatt H. Deminice R. Yoshihara T. Powers S. K. (2019). Mitochondrial Dysfunction Induces Muscle Atrophy during Prolonged Inactivity: A Review of the Causes and Effects. Archives Biochem. Biophy. 662, 49–60. 10.1016/j.abb.2018.11.005 PMC6783132 30452895

[B50] Jaitovich A. Angulo M. Lecuona E. Dada L. A. Welch L. C. Cheng Y. (2015). High CO2 Levels Cause Skeletal Muscle Atrophy via AMP-Activated Kinase (AMPK), FoxO3a Protein, and Muscle-specific Ring Finger Protein 1 (MuRF1). J. Biol. Chem. 290, 9183–9194. 10.1074/jbc.m114.625715 25691571 PMC4423704

[B51] Jones S. E. Maddocks M. Kon S. S. C. Canavan J. L. Nolan C. M. Clark A. L. (2015). Sarcopenia in COPD: Prevalence, Clinical Correlates and Response to Pulmonary Rehabilitation. Thorax 70, 213–218. 10.1136/thoraxjnl-2014-206440 25561517

[B52] Kapchinsky S. Vuda M. Miguez K. Elkrief D. de Souza A. R. Baglole C. J. (2018). Smoke-induced Neuromuscular Junction Degeneration Precedes the Fibre Type Shift and Atrophy in Chronic Obstructive Pulmonary Disease. J. Physiol. 596, 2865–2881. 10.1113/jp275558 29663403 PMC6046075

[B53] Karim A. Muhammad T. Qaisar R. (2021). Prediction of Sarcopenia Using Multiple Biomarkers of Neuromuscular Junction Degeneration in Chronic Obstructive Pulmonary Disease. J. Pers. Med. 11 (9), 919. 10.3390/jpm11090919 34575696 PMC8465187

[B54] Kelly N. A. Hammond K. G. Stec M. J. Bickel C. S. Windham S. T. Tuggle S. C. (2018). Quantification and Characterization of Grouped Type I Myofibers in Human Aging. Muscle Nerve 57, E52–E59. 10.1002/mus.25711 28561923 PMC5711619

[B55] Kitamura M. Kasai A. (2007). Cigarette Smoke as a Trigger for the Dioxin Receptor-Mediated Signaling Pathway. Cancer Lett. 252, 184–194. 10.1016/j.canlet.2006.11.015 17189671

[B56] Konokhova Y. Spendiff S. Jagoe R. T. Aare S. Kapchinsky S. MacMillan N. J. (2016). Failed Upregulation of TFAM Protein and Mitochondrial DNA in Oxidatively Deficient Fibers of Chronic Obstructive Pulmonary Disease Locomotor Muscle. Skelet. muscle 6, 10. 10.1186/s13395-016-0083-9 26893822 PMC4758107

[B57] Korponay T. C. Balnis J. Vincent C. E. Singer D. V. Chopra A. Adam A. P. (2020). High CO2 Downregulates Skeletal Muscle Protein Anabolism via AMP-Activated Protein Kinase α2-mediated Depressed Ribosomal Biogenesis. Am. J. Respir. Cell. Mol. Biol. 62, 74–86. 10.1165/rcmb.2019-0061oc 31264907 PMC6938128

[B58] Kureya Y. Kanazawa H. Ijiri N. Tochino Y. Watanabe T. Asai K. (2016). Down-Regulation of Soluble α-Klotho Is Associated with Reduction in Serum Irisin Levels in Chronic Obstructive Pulmonary Disease. Lung 194, 345–351. 10.1007/s00408-016-9870-7 27140192

[B59] Kuro-o M. Matsumura Y. Aizawa H. Kawaguchi H. Suga T. Utsugi T. (1997). Mutation of the Mouse Klotho Gene Leads to a Syndrome Resembling Ageing. Nature 390, 45–51. 10.1038/36285 9363890

[B60] Lakhdar R. McGuinness D. Drost E. Shiels P. Bastos R. MacNee W. (2018). Role of Accelerated Aging in Limb Muscle Wasting of Patients with COPD. Int. J. Chron. Obstruct. Pulmon. Dis. 13, 1987–1998. 10.2147/copd.s155952 29970961 PMC6022820

[B61] Larsson L. Örlander J. (1984). Skeletal Muscle Morphology, Metabolism and Function in Smokers and Non-smokers. A Study on Smoking-Discordant Monozygous Twins. Acta Physiol. Scand. 120, 343–352. 10.1111/j.1748-1716.1984.tb07394.x 6540035

[B62] Latimer L. E. Constantin-Teodosiu D. Popat B. Constantin D. Houchen-Wolloff L. Bolton C. E. (2021). Whole-body & Muscle Responses to Aerobic Exercise Training and Withdrawal in Ageing & COPD. Eur. Respir. J. 59 (5), 2101507. 10.1183/13993003.01507-2021 PMC9095946 34588196

[B63] Lee J. S. W. Auyeung T.-W. Kwok T. Lau E. M. C. Leung P.-C. Woo J. (2007). Associated Factors and Health Impact of Sarcopenia in Older Chinese Men and Women: a Cross-Sectional Study. Gerontology 53, 404–410. 10.1159/000107355 17700027

[B64] Lee J. Tsogbadrakh B. Yang S. Ryu H. Kang E. Kang M. (2021). Klotho Ameliorates Diabetic Nephropathy via LKB1-AMPK-PGC1α-Mediated Renal Mitochondrial Protection. Biochem. Biophysical Res. Commun. 534, 1040–1046. 10.1016/j.bbrc.2020.10.040 33121684

[B65] Lin B. Bai L. Wang S. Lin H. (2021). The Association of Systemic Interleukin 6 and Interleukin 10 Levels with Sarcopenia in Elderly Patients with Chronic Obstructive Pulmonary Disease. Int. J. Gen. Med. 14, 5893–5902. 10.2147/ijgm.s321229 34566428 PMC8457863

[B66] Liu Y. Bi X. Zhang Y. Wang Y. Ding W. (2020). Mitochondrial dysfunction/NLRP3 Inflammasome axis Contributes to Angiotensin II-Induced Skeletal Muscle Wasting via PPAR-γ. Lab. Invest. 100, 712–726. 10.1038/s41374-019-0355-1 31857693

[B67] Londhe P. Guttridge D. C. (2015). Inflammation Induced Loss of Skeletal Muscle. Bone 80, 131–142. 10.1016/j.bone.2015.03.015 26453502 PMC4600538

[B68] Lustgarten M. S. Fielding R. A. (2017). Metabolites Related to Renal Function, Immune Activation, and Carbamylation Are Associated with Muscle Composition in Older Adults. Exp. Gerontol. 100, 1–10. 10.1016/j.exger.2017.10.003 29030163 PMC6556217

[B69] MacLeod M. Papi A. Contoli M. Beghé B. Celli B. R. Wedzicha J. A. (2021). Chronic Obstructive Pulmonary Disease Exacerbation Fundamentals: Diagnosis, Treatment, Prevention and Disease Impact. Respirology 26, 532–551. 10.1111/resp.14041 33893708

[B70] Maltais F. Decramer M. Casaburi R. Barreiro E. Burelle Y. Debigaré R. (2014). An Official American Thoracic Society/European Respiratory Society Statement: Update on Limb Muscle Dysfunction in Chronic Obstructive Pulmonary Disease. Am. J. Respir. Crit. Care Med. 189, e15–e62. 10.1164/rccm.201402-0373st 24787074 PMC4098112

[B71] Marquis K. Debigaré R. Lacasse Y. LeBlanc P. Jobin J. Carrier G. (2002). Midthigh Muscle Cross-Sectional Area Is a Better Predictor of Mortality Than Body Mass Index in Patients with Chronic Obstructive Pulmonary Disease. Am. J. Respir. Crit. Care Med. 166, 809–813. 10.1164/rccm.2107031 12231489

[B72] Max S. R. Silbergeld E. K. (1987). Skeletal Muscle Glucocorticoid Receptor and Glutamine Synthetase Activity in the Wasting Syndrome in Rats Treated with 2,3,7,8-Tetrachlorodibenzo-P-Dioxin. Toxicol. Appl. Pharmacol. 87, 523–527. 10.1016/0041-008x(87)90258-4 2882621

[B73] McIntosh B. E. Hogenesch J. B. Bradfield C. A. (2010). Mammalian Per-Arnt-Sim Proteins in Environmental Adaptation. Annu. Rev. Physiol. 72, 625–645. 10.1146/annurev-physiol-021909-135922 20148691

[B74] Messi M. L. Li T. Wang Z.-M. Marsh A. P. Nicklas B. Delbono O. (2016). Resistance Training Enhances Skeletal Muscle Innervation without Modifying the Number of Satellite Cells or Their Myofiber Association in Obese Older Adults. Gerona 71, 1273–1280. 10.1093/gerona/glv176 PMC5018557 26447161

[B75] Mezrich J. D. Fechner J. H. Zhang X. Johnson B. P. Burlingham W. J. Bradfield C. A. (2010). An Interaction between Kynurenine and the Aryl Hydrocarbon Receptor Can Generate Regulatory T Cells. J. Immunol. 185, 3190–3198. 10.4049/jimmunol.0903670 20720200 PMC2952546

[B76] Naimi A. I. Bourbeau J. Perrault H. Baril J. Wright-Paradis C. Rossi A. (2011). Altered Mitochondrial Regulation in Quadriceps Muscles of Patients with COPD. Clin. Physiol. Funct. Imaging 31, 124–131. 10.1111/j.1475-097X.2010.00988.x 21091605

[B77] Nebert D. W. (2017). Aryl Hydrocarbon Receptor (AHR): "pioneer Member" of the Basic-Helix/loop/helix Per - Arnt - Sim (bHLH/PAS) Family of "sensors" of Foreign and Endogenous Signals. Prog. Lipid Res. 67, 38–57. 10.1016/j.plipres.2017.06.001 28606467 PMC5568781

[B78] Örlander J. Kiessling K.-H. Larsson L. (1979). Skeletal Muscle Metabolism, Morphology and Function in Sedentary Smokers and Nonsmokers. Acta Physiol. Scand. 107, 39–46. 10.1111/j.1748-1716.1979.tb06440.x 525367

[B79] Ottenheijm C. A. Heunks L. M. Dekhuijzen R. P. (2008). Diaphragm Adaptations in Patients with COPD. Respir. Res. 9, 12. 10.1186/1465-9921-9-12 18218129 PMC2248576

[B80] Pako J. Barta I. Balogh Z. Kerti M. Drozdovszky O. Bikov A. (2017). Assessment of the Anti-aging Klotho Protein in Patients with COPD Undergoing Pulmonary Rehabilitation. COPD J. Chronic Obstr. Pulm. Dis. 14, 176–180. 10.1080/15412555.2016.1272563 28112974

[B81] Patel M. S. Donaldson A. V. Lewis A. Natanek S. A. Lee J. Y. Andersson Y. M. (2016). Klotho and Smoking - an Interplay Influencing the Skeletal Muscle Function Deficits that Occur in COPD. Respir. Med. 113, 50–56. 10.1016/j.rmed.2016.02.004 27021580

[B82] Pérez-Rial S. Barreiro E. Fernández-Aceñero M. J. Fernández-Valle M. E. González-Mangado N. Peces-Barba G. (2020). Early Detection of Skeletal Muscle Bioenergetic Deficit by Magnetic Resonance Spectroscopy in Cigarette Smoke-Exposed Mice. PLoS One 15, e0234606. 10.1371/journal.pone.0234606 32569331 PMC7307759

[B83] Phelps M. Pettan-Brewer C. Ladiges W. Yablonka-Reuveni Z. (2013). Decline in Muscle Strength and Running Endurance in Klotho Deficient C57BL/6 Mice. Biogerontology 14, 729–739. 10.1007/s10522-013-9447-2 24030242 PMC3851892

[B84] Picard M. Godin R. Sinnreich M. Baril J. Bourbeau J. Perrault H. (2008). The Mitochondrial Phenotype of Peripheral Muscle in Chronic Obstructive Pulmonary Disease. Am. J. Respir. Crit. Care Med. 178, 1040–1047. 10.1164/rccm.200807-1005oc 18755922

[B85] Picard M. Hepple R. T. Burelle Y. (2012). Mitochondrial Functional Specialization in Glycolytic and Oxidative Muscle Fibers: Tailoring the Organelle for Optimal Function. Am. J. Physiology-Cell Physiolo. 302, C629–C641. 10.1152/ajpcell.00368.2011 22031602

[B86] Puente-Maestu L. Perez-Parra J. Godoy R. Moreno N. Tejedor A. Gonzalez-Aragoneses F. (2009a). Abnormal Mitochondrial Function in Locomotor and Respiratory Muscles of COPD Patients. Eur. Respir. J. 33, 1045–1052. 10.1183/09031936.00112408 19129279

[B87] Puente-Maestu L. Pérez-Parra J. Godoy R. Moreno N. Tejedor A. Torres A. (2009b). Abnormal Transition Pore Kinetics and Cytochrome C Release in Muscle Mitochondria of Patients with Chronic Obstructive Pulmonary Disease. Am. J. Respir. Cell. Mol. Biol. 40, 746–750. 10.1165/rcmb.2008-0289oc 19011161

[B88] Rak M. Bénit P. Chrétien D. Bouchereau J. Schiff M. El-Khoury R. (2016). Mitochondrial Cytochrome C Oxidase Deficiency. Clin. Sci. (Lond) 130, 393–407. 10.1042/cs20150707 26846578 PMC4948581

[B89] Richardson R. S. Sheldon J. Poole D. C. Hopkins S. R. Ries A. L. Wagner P. D. (1999). Evidence of Skeletal Muscle Metabolic Reserve during Whole Body Exercise in Patients with Chronic Obstructive Pulmonary Disease. Am. J. Respir. Crit. Care Med. 159, 881–885. 10.1164/ajrccm.159.3.9803049 10051266

[B90] Rinaldi M. Maes K. De Vleeschauwer S. Thomas D. Verbeken E. K. Decramer M. (2012). Long-term Nose-Only Cigarette Smoke Exposure Induces Emphysema and Mild Skeletal Muscle Dysfunction in Mice. Dis. Model. Mech. 5, 333–341. 10.1242/dmm.008508 22279084 PMC3339827

[B91] Rocha M. C. Grady J. P. Grünewald A. Vincent A. Dobson P. F. Taylor R. W. (2015). A Novel Immunofluorescent Assay to Investigate Oxidative Phosphorylation Deficiency in Mitochondrial Myopathy: Understanding Mechanisms and Improving Diagnosis. Sci. Rep. 5, 15037. 10.1038/srep15037 26469001 PMC4606788

[B92] Romanello V. Sandri M. (2021). The Connection between the Dynamic Remodeling of the Mitochondrial Network and the Regulation of Muscle Mass. Cell. Mol. Life Sci. 78, 1305–1328. 10.1007/s00018-020-03662-0 33078210 PMC7904552

[B93] Sahu A. Mamiya H. Shinde S. N. Cheikhi A. Winter L. L. Vo N. V. (2018). Age-related Declines in α-Klotho Drive Progenitor Cell Mitochondrial Dysfunction and Impaired Muscle Regeneration. Nat. Commun. 9, 4859. 10.1038/s41467-018-07253-3 30451844 PMC6242898

[B94] Sayed R. K. A. Fernández-Ortiz M. Diaz-Casado M. E. Aranda-Martínez P. Fernández-Martínez J. Guerra-Librero A. (2019). Lack of NLRP3 Inflammasome Activation Reduces Age-dependent Sarcopenia and Mitochondrial Dysfunction, Favoring the Prophylactic Effect of Melatonin. J. Gerontol. A Biol. Sci. Med. Sci. 74, 1699–1708. 10.1093/gerona/glz079 30869745

[B95] Schardong J. Marcolino M. A. Z. Plentz R. D. M. (2018). Muscle Atrophy in Chronic Kidney Disease. Adv. Exp. Med. Biol. 1088, 393–412. 10.1007/978-981-13-1435-3_18 30390262

[B96] Sciacco M. Bonilla E. Schon E. A. DiMauro S. Moraes C. T. (1994). Distribution of Wild-type and Common Deletion Forms of mtDNA in Normal and Respiration-Deficient Muscle Fibers from Patients with Mitochondrial Myopathy. Hum. Mol. Genet. 3, 13–19. 10.1093/hmg/3.1.13 8162014

[B97] Semba R. D. Cappola A. R. Sun K. Bandinelli S. Dalal M. Crasto C. (2012). Relationship of Low Plasma Klotho with Poor Grip Strength in Older Community-Dwelling Adults: the InCHIANTI Study. Eur. J. Appl. Physiol. 112, 1215–1220. 10.1007/s00421-011-2072-3 21769735 PMC3435096

[B98] Semba R. D. Ferrucci L. Sun K. Simonsick E. Turner R. Miljkovic I. (2016). Low Plasma Klotho Concentrations and Decline of Knee Strength in Older Adults. Gerona 71, 103–108. 10.1093/gerona/glv077 PMC4706099 26359247

[B99] Senft A. P. Dalton T. P. Nebert D. W. Genter M. B. Puga A. Hutchinson R. J. (2002). Mitochondrial Reactive Oxygen Production Is Dependent on the Aromatic Hydrocarbon Receptor. Free Radic. Biol. Med. 33, 1268–1278. 10.1016/s0891-5849(02)01014-6 12398935

[B100] Slebos D.-J. van der Toorn M. Bakker S. J. L. Kauffman H. F. (2007). Mitochondrial Dysfunction in COPD Patients with Low Body Mass Index. Eur. Respir. J. 30, 600. author reply 600-601. 10.1183/09031936.00047907 17766639

[B101] Sonjak V. Jacob K. J. Spendiff S. Vuda M. Perez A. Miguez K. (2019b). Reduced Mitochondrial Content, Elevated Reactive Oxygen Species, and Modulation by Denervation in Skeletal Muscle of Prefrail or Frail Elderly Women. J. Gerontol. A Biol. Sci. Med. Sci. 74, 1887–1895. 10.1093/gerona/glz066 30855073

[B102] Sonjak V. Jacob K. Morais J. A. Rivera‐Zengotita M. Spendiff S. Spake C. (2019a). Fidelity of Muscle Fibre Reinnervation Modulates Ageing Muscle Impact in Elderly Women. J. Physiol. 597, 5009–5023. 10.1113/jp278261 31368533

[B103] Spendiff S. Vuda M. Gouspillou G. Aare S. Perez A. Morais J. A. (2016). Denervation Drives Mitochondrial Dysfunction in Skeletal Muscle of Octogenarians. J. Physiol. 594, 7361–7379. 10.1113/jp272487 27619626 PMC5157074

[B104] Szulc P. Duboeuf F. Marchand F. Delmas P. D. (2004). Hormonal and Lifestyle Determinants of Appendicular Skeletal Muscle Mass in Men: the MINOS Study. Am. J. Clin. Nutr. 80, 496–503. 10.1093/ajcn/80.2.496 15277176

[B105] Tényi Á. Cano I. Marabita F. Kiani N. Kalko S. G. Barreiro E. (2018). Network Modules Uncover Mechanisms of Skeletal Muscle Dysfunction in COPD Patients. J. Transl. Med. 16, 34. 10.1186/s12967-018-1405-y 29463285 PMC5819708

[B106] Thome T. Miguez K. Willms A. J. Burke S. K. Chandran V. Souza A. R. (2022). Chronic Aryl Hydrocarbon Receptor Activity Phenocopies Smoking‐induced Skeletal Muscle Impairment. J. cachexia sarcopenia muscle 13, 589–604. 10.1002/jcsm.12826 34725955 PMC8818603

[B107] Thome T. Salyers Z. R. Kumar R. A. Hahn D. Berru F. N. Ferreira L. F. (2019). Uremic Metabolites Impair Skeletal Muscle Mitochondrial Energetics through Disruption of the Electron Transport System and Matrix Dehydrogenase Activity. Am. J. Physiology-Cell Physiolo. 317, C701–C713. 10.1152/ajpcell.00098.2019 PMC6851000 31291144

[B108] Trudzinski F. C. Alqudrah M. Alqudrah M. Omlor A. Zewinger S. Fliser D. (2019). Consequences of Chronic Kidney Disease in Chronic Obstructive Pulmonary Disease. Respir. Res. 20, 151. 10.1186/s12931-019-1107-x 31299972 PMC6626422

[B109] van de Bool C. Gosker H. R. van den Borst B. Op den Kamp C. M. Slot I. G. M. Schols A. M. W. J. (2016). Muscle Quality Is More Impaired in Sarcopenic Patients with Chronic Obstructive Pulmonary Disease. J. Am. Med. Dir. Assoc. 17, 415–420. 10.1016/j.jamda.2015.12.094 26848065

[B110] van den Borst B. Koster A. Yu B. Gosker H. R. Meibohm B. Bauer D. C. (2011). Is Age-Related Decline in Lean Mass and Physical Function Accelerated by Obstructive Lung Disease or Smoking? Thorax 66, 961–969. 10.1136/thoraxjnl-2011-200010 21724748 PMC3285455

[B111] van den Borst B. Slot I. G. M. Hellwig V. A. C. V. Vosse B. A. H. Kelders M. C. J. M. Barreiro E. (2013). Loss of Quadriceps Muscle Oxidative Phenotype and Decreased Endurance in Patients with Mild-To-Moderate COPD. J. Appl. Physiolo. 114, 1319–1328. 10.1152/japplphysiol.00508.2012 22815389

[B112] Wang X. H. Zhang L. Mitch W. E. LeDoux J. M. Hu J. Du J. (2010). Caspase-3 Cleaves Specific 19 S Proteasome Subunits in Skeletal Muscle Stimulating Proteasome Activity. J. Biol. Chem. 285, 21249–21257. 10.1074/jbc.m109.041707 20424172 PMC2898444

[B113] Webster J. M. Kempen L. J. A. P. Hardy R. S. Langen R. C. J. (2020). Inflammation and Skeletal Muscle Wasting during Cachexia. Front. Physiol. 11, 597675. 10.3389/fphys.2020.597675 33329046 PMC7710765

[B114] Westbrook R. Chung T. Lovett J. Ward C. Joca H. Yang H. (2020). Kynurenines Link Chronic Inflammation to Functional Decline and Physical Frailty. JCI Insight 5 (16), e136091. 10.1172/jci.insight.136091 PMC7455140 32814718

[B115] Wüst R. C. I. Morse C. I. de Haan A. Rittweger J. Jones D. A. Degens H. (2008). Skeletal Muscle Properties and Fatigue Resistance in Relation to Smoking History. Eur. J. Appl. Physiol. 104, 103–110. 10.1007/s00421-008-0792-9 18560879 PMC2480601

[B116] Xiong J. Le Y. Rao Y. Zhou L. Hu Y. Guo S. (2021). RANKL Mediates Muscle Atrophy and Dysfunction in a Cigarette Smoke-Induced Model of Chronic Obstructive Pulmonary Disease. Am. J. Respir. Cell. Mol. Biol. 64, 617–628. 10.1165/rcmb.2020-0449oc 33689672

[B117] Yi S.-W. Hong J.-S. Ohrr H. Yi J.-J. (2014). Agent Orange Exposure and Disease Prevalence in Korean Vietnam Veterans: the Korean Veterans Health Study. Environ. Res. 133, 56–65. 10.1016/j.envres.2014.04.027 24906069

[B118] Zhou B. Wang X. Li F. Wang Y. Yang L. Zhen X. (2017). Mitochondrial Activity and Oxidative Stress Functions Are Influenced by the Activation of AhR-Induced CYP1A1 Overexpression in Cardiomyocytes. Mol. Med. Rep. 16, 174–180. 10.3892/mmr.2017.6580 28498411 PMC5482149

